# Key signaling networks are dysregulated in patients with the adipose tissue disorder, lipedema

**DOI:** 10.1038/s41366-021-01002-1

**Published:** 2021-11-11

**Authors:** Musarat Ishaq, Nadeeka Bandara, Steven Morgan, Cameron Nowell, Ahmad M. Mehdi, Ruqian Lyu, Davis McCarthy, Dovile Anderson, Darren J. Creek, Marc G. Achen, Ramin Shayan, Tara Karnezis

**Affiliations:** 1 Lymphatic, Adipose and Regenerative Medicine Laboratory, O’Brien Institute Department, St Vincent’s Institute of Medical Research, Fitzroy, VIC 3065 Australia; 2 Department of Medicine, St Vincent’s Hospital, University of Melbourne, Fitzroy, VIC 3065 Australia; 3 Monash Institute of Pharmaceutical Sciences, Monash University, Parkville, VIC 3052 Australia; 4 Diamantia Institute, Faculty of Medicine, The University of Queensland, St Lucia, QLD 4067 Australia; 5 Bioinformatics and Cellular Genomics, St. Vincent’s Institute of Medical Research, Fitzroy, VIC 3065 Australia

**Keywords:** Obesity, Obesity

## Abstract

**Objectives:**

Lipedema, a poorly understood chronic disease of adipose hyper-deposition, is often mistaken for obesity and causes significant impairment to mobility and quality-of-life. To identify molecular mechanisms underpinning lipedema, we employed comprehensive omics-based comparative analyses of whole tissue, adipocyte precursors (adipose-derived stem cells (ADSCs)), and adipocytes from patients with or without lipedema.

**Methods:**

We compared whole-tissues, ADSCs, and adipocytes from body mass index–matched lipedema (*n* = 14) and unaffected (*n* = 10) patients using comprehensive global lipidomic and metabolomic analyses, transcriptional profiling, and functional assays.

**Results:**

Transcriptional profiling revealed >4400 significant differences in lipedema tissue, with altered levels of mRNAs involved in critical signaling and cell function-regulating pathways (e.g., lipid metabolism and cell-cycle/proliferation). Functional assays showed accelerated ADSC proliferation and differentiation in lipedema. Profiling lipedema adipocytes revealed >900 changes in lipid composition and >600 differentially altered metabolites. Transcriptional profiling of lipedema ADSCs and non-lipedema ADSCs revealed significant differential expression of >3400 genes including some involved in extracellular matrix and cell-cycle/proliferation signaling pathways. One upregulated gene in lipedema ADSCs, *Bub1*, encodes a cell-cycle regulator, central to the kinetochore complex, which regulates several histone proteins involved in cell proliferation. Downstream signaling analysis of lipedema ADSCs demonstrated enhanced activation of histone H2A, a key cell proliferation driver and Bub1 target. Critically, hyperproliferation exhibited by lipedema ADSCs was inhibited by the small molecule Bub1 inhibitor 2OH-BNPP1 and by CRISPR/Cas9-mediated *Bub1* gene depletion.

**Conclusion:**

We found significant differences in gene expression, and lipid and metabolite profiles, in tissue, ADSCs, and adipocytes from lipedema patients compared to non-affected controls. Functional assays demonstrated that dysregulated Bub1 signaling drives increased proliferation of lipedema ADSCs, suggesting a potential mechanism for enhanced adipogenesis in lipedema. Importantly, our characterization of signaling networks driving lipedema identifies potential molecular targets, including Bub1, for novel lipedema therapeutics.

## Introduction

Lipedema is a poorly understood, chronic disease characterized by bilaterally symmetrical deposition of abnormal adipose tissue, predominantly in the legs and variably in the arms [1, 2]. The classification of lipedema is based on the distribution of adipose tissue and on the severity of the disease (Stages I, II, III, and IV) [3] (Supplementary Fig. 1a). Most metabolic disorders relating to adipose tissue are associated with white adipose tissue (WAT), and dysfunction of its endocrine activities [4]. Excessive accumulation of WAT in abdominal organs (visceral adipose tissue) and subcutaneous regions disrupts metabolic homeostasis and likely drives metabolic disorders [4, 5]. In lipedema, patients accumulate excessive amounts of subcutaneous WAT, predominately in the legs [2].

Lipedema mostly affects women, typically starting during stages of hormonal change such as puberty, pregnancy, in vitro fertilization, or menopause. Symptoms include extreme leg sensitivity, hyperadiposity, swelling, pain, bruising, and joint complications (hyper-flexibility and later arthritis), resulting in impaired mobility and body image. Lipedema is often confused with obesity, lymphedema, lipodystrophies, and other fat disorders [1] and currently is diagnosed clinically. Palpation and intraoperative examination of lipedema fat reveals a nodular structure and consistency, in contrast to normal fat that is smooth and soft [6–8]. There is no cure for lipedema, and treatments are limited to invasive liposuction or excisional surgery to control symptoms and enhance mobility and esthetics. The etiology of lipedema is unknown, although clinical observations suggest genetic inheritance, hormonal influences, dilated blood vessels and lymphatics, and inflammation [3]. Despite a relatively high incidence of lipedema [6–8], molecular or genetic data to explain the pathogenesis of the disease are lacking [9–12]. To date, elucidation of the molecular mechanisms underlying lipedema has been hampered by the lack of lipedema animal models.

In adipose disorders, fat typically expands by increasing either the size or number of adipocytes [13]. While adipocytes are unable to divide, they can increase in size to accommodate greater amounts of lipid volume [13]. Adipocytes form via de novo differentiation from adipose-derived stem cells (ADSCs) upon adipogenic induction [13]. Increased ADSC numbers, therefore, can lead to increased differentiation into adipocytes and enhanced adipose deposition in pathologic fat disorders. It has been proposed that adipocytes increase in both size and number in lipedema; however, the molecular mechanisms by which ADSCs undergo proliferation or differentiation to adipocytes, resulting in excessive accumulation of lipedema adipose tissue, are poorly understood [13–16].

Here, we report on comprehensive comparative multi-omics analyses of adipose tissue, ADSCs, and adipocytes from lipedema and non-lipedema patients, which emphasize the profoundly distinct nature of lipedema and non-lipedema adipose tissue. Importantly, our omics analyses led us to identify signaling events central to enhanced ADSC proliferation in lipedema, from which we validate a gene candidate and derive a proof-of-principle therapeutic intervention. Our findings substantially improve understanding of the molecular mechanisms underpinning lipedema and will be useful for identifying disease biomarkers and targets for future therapeutics with which to combat this devastating disease.

## Results

### Lipedema adipose tissues show distinct gene signatures and ADSC densities to normal fat

**Fig. 1:**
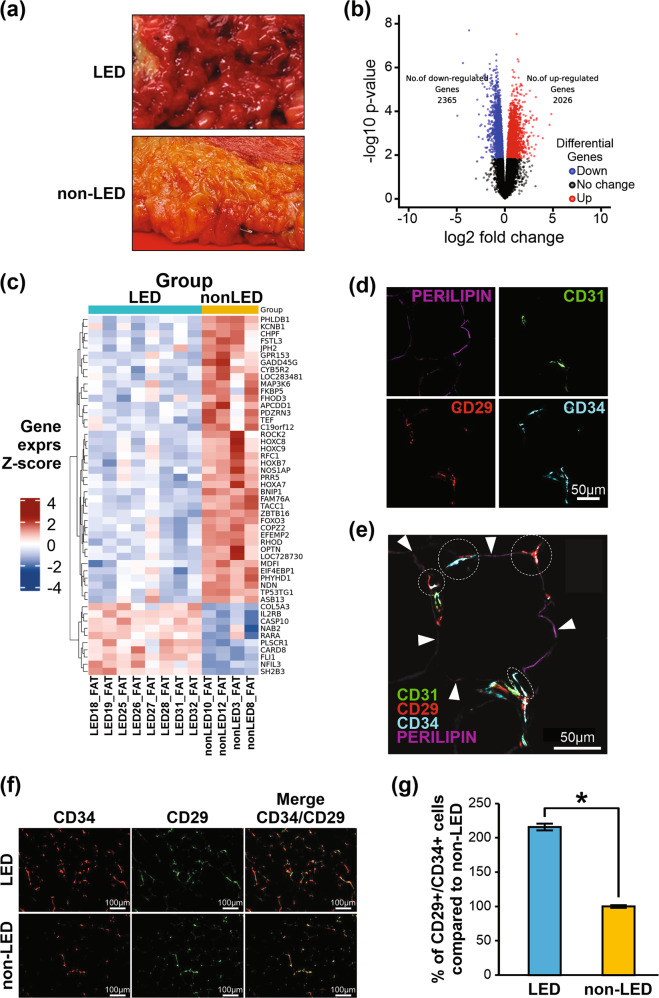
Transcriptional profiling of adipose tissue from lipedema (LED) and non-lipedema (non-LED) patients. **a** Lipedema and non-lipedema adipose tissue after surgery. **b** Volcano plot of significantly differentially expressed genes (adj. *p* < 0.05). **c** Heatmap of hierarchical clustering showing expression patterns of top 50 differentially expressed genes ranked by topTreat from limma. *Z*-score fold-change value: red indicates upregulation and blue indicates downregulation (lipedema *n* = 8; non-lipedema *n* = 4). **d** CD29, CD34, CD31, and perilipin staining of adipocytes from lipedema tissue. **e** Perilipin-stained adipocyte (purple) from lipedema adipose tissue with two small blood vessels (CD31^+^(green)), and ADSCs (CD34^+^(blue) CD29^+^(red)). Circles indicate CD29^+^/CD34^+^ and CD31^–^ co-localized cells. White arrowheads indicate perilipin staining (purple) of adipocyte. **f**, **g** Quantification of CD29^+^/CD34^+^ cells by ImageJ from lipedema and non-lipedema adipose tissue (*n* = 3, 6 random fields were quantified).

Recently, it was demonstrated that lipedema adipose tissue has larger adipocytes, increased macrophage density and increased dermal blood and lymphatic vessels compared to normal fat [17]. Whether the large amounts of adipose tissue accumulated in lipedema (Supplementary Fig. 1a) differ from non-lipedema fat at the cellular and molecular levels, however, remains to be elucidated. To address this, adipose tissues surgically excised by the same surgeon (RS) from equivalent anatomical sites in lipedema patients and gender-, health-, age-, and BMI-matched (Supplementary Table 1) non-lipedema patients undergoing elective debulking surgery were compared. Macroscopic examination showed lipedema fat to be harder and more fibrotic and nodular than smooth, softer normal fat (Fig. 1a).

To understand the molecular mechanisms underlying these macroscopic changes, we undertook transcriptome profiling to characterize and quantify the transcriptomes of lipedema and non-lipedema adipose tissue. Principle component analysis (PCA) indicated separation of lipedema from non-lipedema samples and showed clear differences between their transcriptomic profiles (Supplementary Fig. 1b), and differential gene expression analysis showed 4391 genes to be significantly differentially expressed (Fig. 1b and Supplementary Table 2). Hierarchical clustering of the top genes showed distinct subsets of lipedema and non-lipedema genes (Fig. 1c), with gene ontology (GO) annotation and pathway enrichment analysis highlighting cell proliferation, lipid metabolism, cell adhesion, inflammation, and immunity pathways (Supplementary Fig. 1c–e). Highly differential gene expression that biologically favored enhanced proliferation in lipedema involved the cell-cycle regulation- and proliferation-related genes *ZIC1*, *UGT1A7*, *GREM1*, *TRIM67*, *Bub1*, and *HOTAIR*. Overall, global gene expression data and pathway enrichment analyses showed significant alterations in the molecular signature of lipedema tissue, with a gene expression profile favoring adipose hyperproliferation, fibrosis, and inflammation, which are consistent with key clinical features of lipedema.

To evaluate potential cells within lipedema fat that may contribute to the hypertrophy detected by our tissue transcriptomics, we focused on ADSCs—critical effector cells in fat. ADSCs play diverse roles in immune-modulation, angiogenesis, apoptosis, and differentiation [18] and are precursors of adipocytes, the major cell constituent of adipose tissue. Of described ADSC markers [19], CD29 and CD34 cell surface markers were chosen to define ADSCs [20, 21]. To visualize the ADSCs within the perivascular niche, we performed multiplex immunostaining with the adipocyte marker perilipin, the endothelial cell marker CD31 and CD34 and CD29. We identified perilipin^+^ adipocytes in tissue colocalizing with CD31^+^ endothelial cells and CD29^+^/CD34^+^ ADSCs (Fig. 1d, e). Next, we quantified the numbers of CD29^+^/CD34^+^ cells in tissue specimens and found that lipedema adipose tissue showed greater numbers of CD29^+^/CD34^+^ ADSCs compared to control tissue (Fig. 1f, g), suggesting a greater propensity to generate fat when stimulated. Together, these results indicate that the adipose tissue microenvironment in lipedema differs significantly from that in non-lipedema patients, both in terms of its abundance of ADSCs and gene expression profiles.

### ADSCs and adipocytes in lipedema tissue are functionally distinct from those in obese fat

**Fig. 2:**
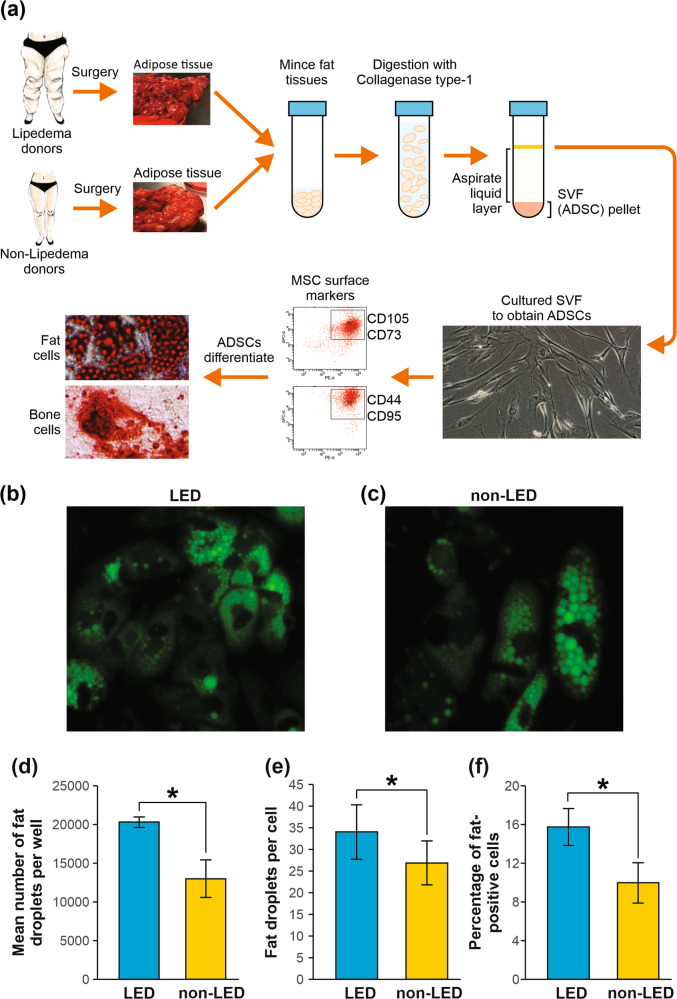
Isolation and characterization of lipedema and non-lipedema ADSCs from adipose tissue. **a** Schematic showing isolation of ADSCs from adipose tissue. **b**, **c** ADSCs were isolated from lipedema and non-lipedema adipose tissue, grown in adipogenic differentiation media for 14 days and analyzed with Bodipy staining. Fat droplets stained green with Bodipy. **d**–**f** For adipogenic differentiation assays, lipedema ADSCs and non-lipedema ADSCs (40,000 cells/well in 48 well plate) were cultured for 24 h in growth media, followed by incubation in adipogenic differentiation media for 14 days. Adipogenic differentiation was then detected by Bodipy staining of neutral lipids in differentiated cells. Graphs represent quantification of fat droplets per well (**d**), fat droplets per cell (**e**), and percentage of fat-positive cells (**f**) (*n* = 3, mean ± SEM of three independent experiments, *p < 0.05, Student’s *t* test).

Having demonstrated key differences in lipedema tissues, including higher numbers of CD29^+^/CD34^+^ ADSCs, we next focused on the role of these ADSCs, as stem cells play key roles in many diseases, and ADSCs are precursors to adipocytes that may underpin the pathogenesis of lipedema. We employed a method of ADSC enrichment from adipose tissues, enabling us to study these cells in cell-based assays (Fig. 2a). Single-cell suspensions were prepared from lipedema and control tissues and the ADSC-enriched stromal vascular fraction assessed by flow cytometry. We validated that ADSCs from both patient groups displayed characteristic spindle morphology and expression of ADSC markers [22] (Supplementary Fig. 2a–c).

To assess the adipogenic properties of the ADSC populations, we performed differentiation studies of ADSCs to adipocytes. When stimulated to differentiate to adipocytes in vitro, ADSCs functionally resemble adipocytes in several key aspects, such as lipid accumulation [20, 21, 23], allowing differentiated ADSCs to be used as a surrogate in vitro adipogenesis model for physiological and functional investigations. Adipocyte differentiation of our enriched ADSCs was verified by staining for lipid droplets (consisting mainly of neutral lipids, triglyceride, and cholesterol esters [24]). BODIPY-lipid staining revealed that the number of fat droplets, percentage of fat-positive cells, and number of fat droplets/cell were significantly increased in lipedema adipocytes compared to controls (Fig. 2b–f). These findings suggest that lipedema ADSC populations differ from non-lipedema ADSCs in terms of growth and differentiation capacity, which may play a critical role in the pathogenesis of lipedema.

### Global lipidomic analysis showed altered lipid composition in lipedema adipocytes

**Fig. 3:**
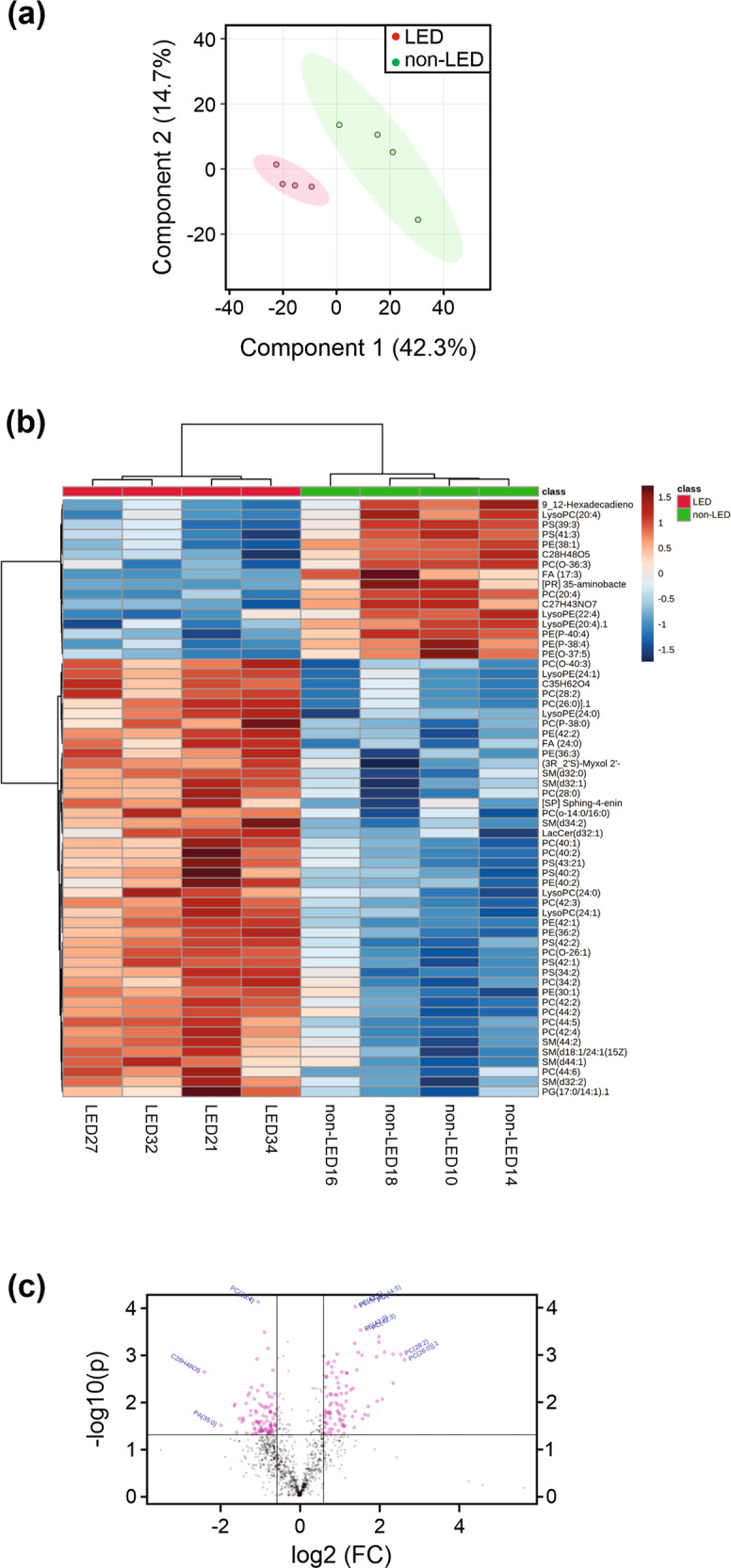
Comparison of lipid content in adipocytes from lipedema and non-lipedema patients. Lipedema (LED) ADSCs and non-lipedema (non-LED) ADSCs were treated with adipogenic differentiation medium for 14 days. Adipocytes were then collected and analyzed for differences in lipid content. **a** Supervised partial least squares discriminant analysis (PLS-DA) plot of four biological replicates from lipedema and non-lipedema adipocytes show clear separation of two different classes of metabolites. **b** Hierarchical clustering analysis of the 50 lipids with greatest differences in abundance between lipedema and non-lipedema adipocytes. **c** Volcano plot showing highly different lipid contents.

After showing that the lipid storage architecture within adipocytes differed in differentiated lipodema adipocytes, we next wanted to assess their lipid composition. Adipocyte lipid composition has been shown to contribute to several clinical disorders including obesity and cancer [25–27]. Therefore, to characterize the lipid profiles of adipocytes in lipedema, we used a non-targeted lipidomics approach, focusing on the profile of low molecular weight (*m/z* 300–3000) ionizable lipid molecules, and multivariate statistics to compare the lipid molecules in lipedema adipocytes against controls. Liquid chromatography mass spectrometry (LCMS) data acquisition identified 928 putative lipid species based on accurate mass [26, 27]. PCA analysis revealed distinct clusters for each of the adipocyte groups, suggesting a unique lipedema adipocyte lipidomic signature (Fig. 3a and Supplementary Fig. 3a). Next, the variable importance in projection (VIP) scores from a PLS-DA analysis were used to rank the most significant lipid differences. The leading significantly increased lipids in lipedema included glycerophospholipids (GPLs) LPE(24:1), PC(28:2), PC(26:0), PE(42:2), PE(42:1), LysoPC(24:1), and PC(42:3) (Supplementary Fig. 3b). To further characterize the lipid profiles of lipedema adipocytes, hierarchical clustering analysis was performed, which identified the altered lipids in lipedema (Fig. 3b). This analysis showed separation of the lipidomic signatures of lipedema from non-lipedema profiles, and after global characterization, we further filtered the 928 original lipid species detected to 112 significantly differing species between the groups. The lipid species of greatest differences between the groups included sphingolipids and GPLs and to a lesser extent, gangliosides, fatty acyls, and glycerolipids (Supplementary Fig. 3c and Supplementary Table 3). Utilizing a volcano plot to elucidate the lipid metabolites that are present at different levels in lipedema versus non-lipedema adipocytes showed a global effect (Fig. 3c). Lipids across all major lipid classes seem to be affected in lipedema adipocytes as can be seen from a heatmap of lipid classes (Supplementary Fig. 3d). These experiments demonstrated that, globally, lipids were significantly altered in adipocytes differentiated from the ADSCs of lipedema patients compared to non-lipedema patients.

### Global metabolic profiles are significantly altered in patients with lipedema

Previous studies showed that patients with adipose disorders but relatively normal metabolic status have lower mortality rates than patients with abnormal metabolic status [28]; however, the reason for this is unknown. Therefore, having demonstrated abnormalities in the lipidomic profile of lipedema adipocytes, we undertook a metabolomics analysis to profile the small endogenous molecules or metabolites in lipedema. A metabolic comparison of adipocytes was undertaken, and PCA showed separation between lipedema and non-lipedema groups (four biological replicates) (Supplementary Fig. 4a). Of the 640 distinct metabolites putatively identified in lipedema adipocytes, the most highly represented metabolite classes related to amino acid and carbohydrate metabolism (Supplementary Fig. 4b–d). VIP scores from a PLS-DA analysis were used to rank the most significant metabolite differences (Supplementary Fig. 4e). Pathway analysis suggested that perturbations in lipedema included pathways relating to amino acid metabolism (lysine biosynthesis and glutamate metabolism), peptides, and GLPs (Supplementary Table 4). It has been shown previously that metabolome imbalances result in adipocyte hypertrophy and/or hyperplasia that lead to fat disorders [29]. Taken together, our results indicate that the metabolites most deranged in lipedema may therefore critically contribute to disease pathogenesis.

### ADSCs from lipedema adipose tissue display an enhanced proliferative state compared to those from BMI-matched patients

**Fig. 4:**
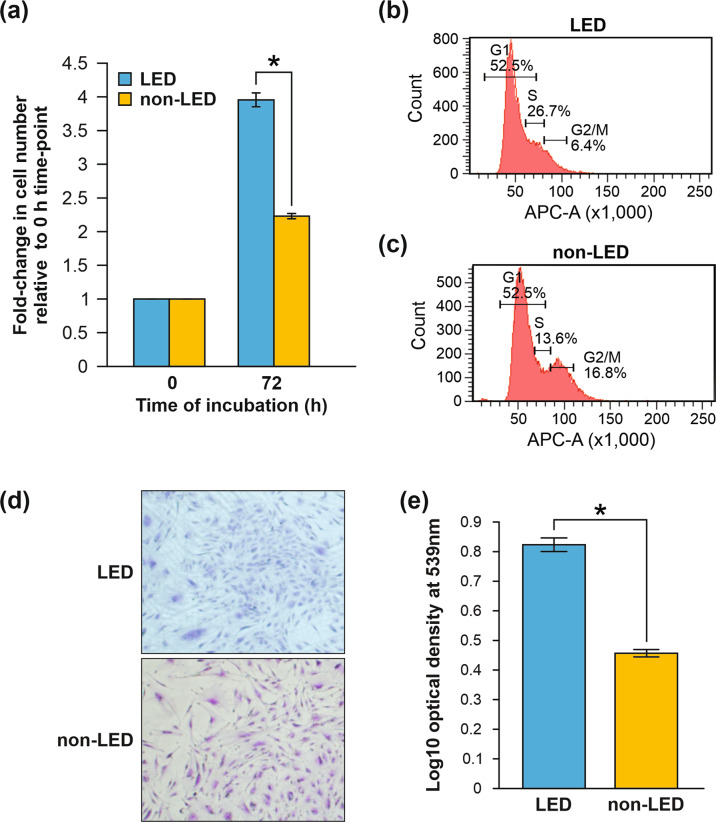
Lipedema ADSCs exhibit enhanced proliferative potential compared to non-lipedema ADSCs. **a** Lipedema (LED) ADSCs and non-lipedema (non-LED) ADSCs were cultured in 96-well plates (3000 cells/well) for 72 h in growth media and cell growth was monitored by time-lapse microscopy (*n* = 6, mean ± SEM of two independent experiments, *p < 0.05, Student’s *t* test). **b**, **c** For cell-cycle assays, lipedema ADSCs (**b**) and non-lipedema ADSCs (**c**) were cultured in growth media for 48 h and stained with FXCycle Cell Cycle Assay Kit and analyzed by flow cytometry. **d**, **e** Clonogenicity potential of ADSCs as assessed by crystal violet staining of colony formation units (CFU). The stain was dissolved with 0.5% sodium dodecyl sulfate and absorbance was measured at 539 nm. Graph shows optical density for lipedema and non-lipedema (*n* = 3, mean ± SEM, *p < 0.05, Student’s *t* test).

Having shown a gene expression profile in lipedema tissue that favors hyperproliferation, we evaluated the proliferative potential of lipedema ADSCs using proliferation assays to monitor their growth rates. Time-lapse microscopy showed that ADSCs from lipedema patients proliferated faster than control ADSCs (Fig. 4a). It is well documented that an accumulation of cells in S phase indicates cellular hyperproliferation and activation of the intra S phase checkpoint [30, 31]. In order to study cell division and elucidate the mechanisms by which lipedema ADSCs become hyperproliferative, cell-cycle analysis was performed. After synchronizing the ADSC groups by growth factor starvation to drive cells into the same phase (G0), complete medium was added to stimulate the cells to initiate proliferation. Results showed that 72 h after the addition of complete growth media, a higher percentage of lipedema ADSCs was in S phase (26.7%) compared to non-lipedema ADSCs (13.6%) (Fig. 4b, c). Finally, a colony formation unit (CFU) assay demonstrated an enhanced colony formation capacity of the hyperproliferative lipedema ADSCs (Fig. 4d, e). Together, these findings underscored the enhanced self-renewal and proliferative potential of lipedema ADSCs that may combine to drive adipose proliferation in lipedema.

### Altered cell-cycle pathways drive adipogenesis and proliferation in lipedema ADSCs

**Fig. 5:**
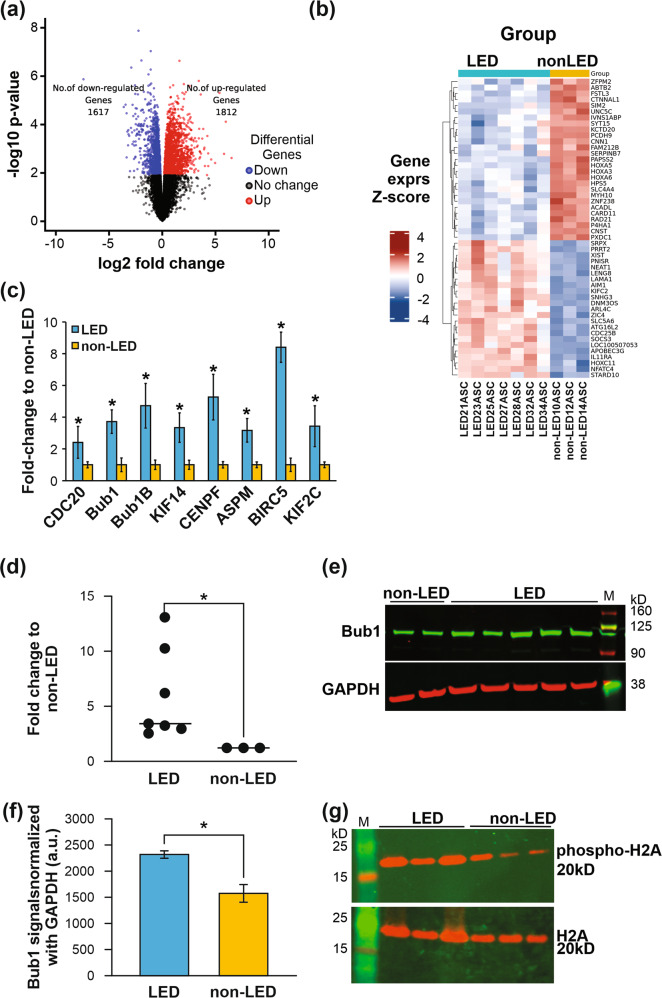
Cell-cycle genes that are differentially expressed in lipedema enhance proliferative capacity of ADSCs. **a** RNA-seq analyses of lipedema (LED) ADSCs from seven patients (LED21, 23, 25, 27, 28, 32, 34) and of non-lipedema (non-LED) ADSCs from three patients (non-LED10, 12, 14) indicate differences between lipedema ADSC and non-lipedema ADSC transcript signatures. Volcano plot analysis of significantly differentially expressed genes (adj. *p* < 0.05) showed that 1617 genes were downregulated and 1812 genes were upregulated in lipedema ADSCs compared to non-lipedema ADSCs. **b** Heatmap of hierarchical clustering showing representative gene expression patterns. Top 50 differentially expressed genes (ranked by topTreat from limma) in lipedema ADSCs compared to non-lipedema ADSCs. *Z*-score fold-change value: red indicates upregulation and blue indicates downregulation. **c** Differentially expressed genes involved in mitotic spindle checkpoint pathways of cell cycle were validated by quantitative qRT-PCR (*n* = 3, mean ± SEM of two independent experiments, *p < 0.05, Student’s *t* test). **d** Bub1 mRNA was quantified by qRT-PCR in lipedema ADSCs isolated from seven patients and non-lipedema ADSCs from three patients (*p < 0.05, Student’s *t* test). **e** Bub1 protein from lipedema ADSCs (LED21, 23, 27, 32, 34) and non-lipedema ADSCs (non-LED10, 12) was analyzed by western blotting. **f** Graph represents relative expression levels of Bub1 protein, as assessed by western blotting, normalized to GAPDH levels (mean ± SEM of two independent experiments, *p < 0.05, Student’s *t* test). **g** Levels of Histone H2A (H2A) (lower), a target of Bub1, and of phosphorylated H2A (upper), were analyzed in lipedema ADSCs (LED21, 27, 34) and non-lipedema ADSCs (non-LED10, 12, 14) by Western blotting.

We demonstrated transcriptomic differences in whole lipedema tissue and a hyperproliferative profile and functional behavior of lipedema ADSCs. To understand potential mechanism, we next explored the transcriptome of lipedema ADSCs themselves. RNA-seq followed by MDS and PCA analyses revealed clear separation, indicating different gene signatures, between lipedema and control ADSC transcriptomes (Supplementary Fig. 5a). In keeping with whole tissue profiles, differential expression analysis showed 3429 genes to be significantly differentially expressed between lipedema and control ADSCs (Fig. 5a, b and Supplementary Table 5). These data were further analyzed using the signaling pathway and GO terms cell-cycle, cell proliferation, cell division, and mitotic cell-cycle processes (Supplementary Fig. 5b). Cell-cycle mitotic pathways were among those most significantly differentially expressed (>50 genes) including cell-cycle genes that regulate and maintain the mitotic spindle checkpoint (Supplementary Fig. 5c, d). Significantly expressed genes were selected from the RNA-seq data set for validation by qRT-PCR. Among these genes, eight differentially expressed genes (*Bub1*, *Bub1B*, *CDC20*, *CENPF*, *ASPM*, *BIRC5*, *KIF2C*, and *KIF14*) were validated (Fig. 5c). These findings suggested that cell-cycle genes involved in regulating cell growth and proliferation are dysregulated in lipedema ADSCs and may contribute to the increased adipocyte number, and maldistribution and accumulation of dystrophic fat in lipedema.

### Bub1 hyper-activation drives enhanced proliferation of ADSCs from lipedema patients

Of our validated candidates, Bub1 was selected for further study because: (1) Bub1 mRNA was significantly upregulated in both lipedema whole tissue and ADSCs; (2) Bub1 overexpression is associated with hyperproliferation and dysregulation of key processes in several cancers [32–37]; and (3) Bub1 is a mitotic checkpoint protein identified as a potential therapeutic target in cancer stem cells [32, 36, 38–43]. From a mechanistic perspective, Bub1 plays a role in transcriptional activation during G1/S transition by phosphorylating histone H2A [44], and knockdown of Bub1 by siRNA can arrest cells in the G1/S phase [45]. Conversely, overexpression of Bub1 results in increased cell proliferation and chromosomal instability via hyper-activation of histone H2A [44].

Our finding that Bub1 mRNA is overexpressed in lipedema ADSCs suggested to us that Bub1 may play a similar role in lipedema as it does in cancer (i.e., driving excessive cell proliferation), and could therefore be a potential therapeutic target. Consequently, we sought to analyze Bub1 transcript levels in ADSCs from lipedema and non-lipedema patients by qRT-PCR, which demonstrated that Bub1 mRNA was significantly upregulated in lipedema ADSC populations, compared to controls (Fig. 5d). Bub1 protein was also significantly upregulated in lipedema ADSCs as assessed by Western blotting (Fig. 5e, f). Having established upregulation of *Bub1* gene expression by numerous methods, we analyzed phosphorylation of histone H2A, a downstream signaling target of Bub1 integral to Bub1-driven cell proliferation. Western blotting revealed a greater level of phosphorylated histone H2A in ADSCs from lipedema patients compared to controls (Fig. 5g), suggesting that ADSCs are “primed” to proliferate in lipedema.

**Fig. 6:**
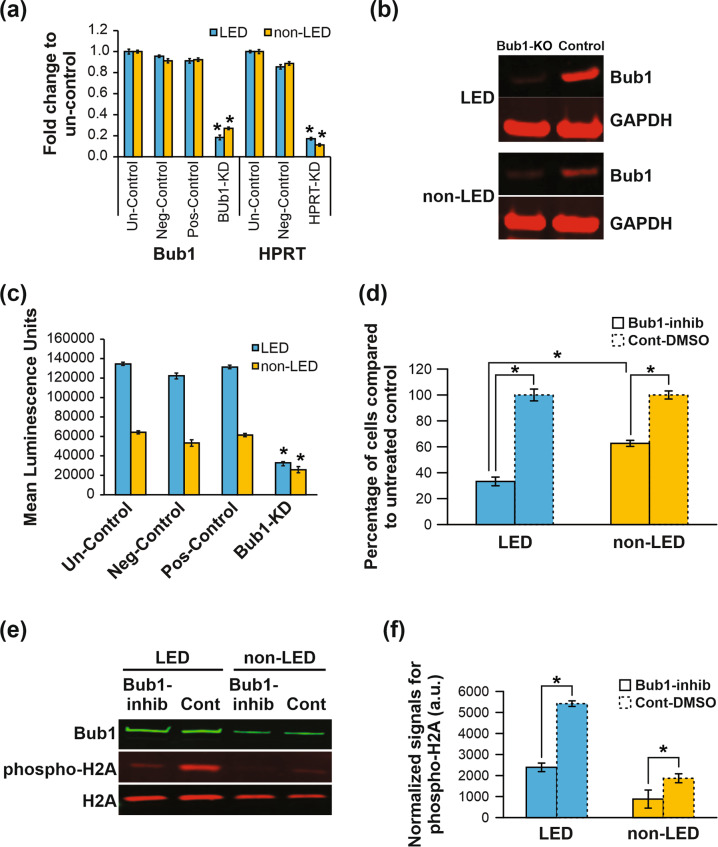
Bub1 drives enhanced proliferation of lipedema ADSCs. **a** CRISPR/Cas9 lentiviral vectors that express gRNA for *Bub1* gene (Bub1-KD), gRNA for the *HPRT* gene as positive control (pos-control), and scrambled gRNA as negative control (neg-control) were used in combination with lentiviral Cas9 to infect lipedema ADSCs and non-lipedema ADSCs with or without any treatment of viral vectors (untreated control; un-control). After 7 days of infection, ADSCs were used to extract RNA, and qRT-PCR analysis was performed to quantify Bub1 and HPRT transcripts. **b** Western blotting to quantify Bub1 protein in lipedema ADSCs and non-lipedema ADSCs after gene removal with CRISPR/Cas9 lentiviral vector expressing gRNA for the *Bub1* gene. GAPDH was used as loading control. **c** Proliferation of ADSCs was measured following gene depletion of Bub1 (mean ± SEM of three independent experiments, *p < 0.05, Student’s *t* test). **d** Lipedema ADSCs and non-lipedema ADSCs were cultured in growth medium with or without Bub1 inhibitor (2OH-BNPP1, 25 μM) or DMSO. Cell proliferation was measured by DAPI staining after 3 days. Relative levels of proliferation for treated and control cells are shown (mean ± SEM of three independent experiments, *p < 0.05, Student’s *t* test). **e** Treatment of ADSCs with 2OH-BNPP1 inhibited phosphorylation of Histone H2A. Western blotting was performed to quantify phosphorylated H2A in lipedema ADSCs and non-lipedema ADSCs after treatment with or without 2OH-BNPP1. **f** Quantification of phosphorylated H2A normalized with levels of total H2A protein (mean ± SEM of two independent experiments, *p < 0.05, Student’s *t* test).

To ascertain the effects of restricting Bub1 in lipedema ADSCs, we employed a CRISPR/Cas9-mediated lentiviral system to knockdown Bub1 and a small molecule Bub1 inhibitor. Using qRT-PCR analysis, we observed an approximately 80% reduction of Bub1 mRNA levels in Bub1-depleted ADSCs compared to negative controls (Fig. 6a). Furthermore, Western blot confirmed decreased Bub1 protein in Bub1-knockdown ADSCs (Fig. 6b). As expected, knockdown of *Bub1* gene decreased proliferation of both lipedema and non-lipedema ADSCs; however, the reduction in proliferation was significantly more pronounced in lipedema ADSCs (Fig. 6c). To explore a pharmacological approach for inhibiting Bub1 in lipedema ADSCs, we next employed the small molecule 2OH-BNPP1, which specifically inhibits the Serine/Threonine kinase activity of Bub1 [46]. We found that 2OH-BNPP1 significantly and profoundly restricted proliferation of lipedema ADSCs by approximately 77%, after 3 days of treatment, whereas inhibition of non-lipedema ADSC proliferation was more modest at approximately 40% (Fig. 6d). We also tested the effect of 2OH-BNPP on the cell cycle in lipedema and non-lipedema ADSCs. As expected, cell-cycle analysis showed that ADSC treatment with 2OH-BNPP resulted in more cells arrested at G1/S phase for both lipedema and non-lipedema ADSCs, with lipedema ADSCs showing a more significantly pronounced effect (Supplementary Fig. 6a, b), suggesting that the hyperproliferative state of lipodema ADSCs makes them more sensitive to cell-cycle inhibitor agents than the non-lipedema controls.

As it has been shown that Bub1 enhances cell proliferation by phosphorylating threonine 120 (T120) of histone H2A [33, 46–48], we finally tested the effect of 2OH-BNPP1 on the activation of histone H2A, which is downstream of Bub1. Treatment of both ADSC groups with 2OH-BNPP1 revealed marked reduction of histone H2A phosphorylation at T120 (Fig. 6e, f), a novel and important finding that suggested that the effect of Bub1 in lipedema is mediated by its capacity to phosphorylate downstream targets such as histone H2A.

## Discussion

Despite increasing clinical recognition of lipedema, mechanisms underlying this problematic disease remain unknown. To explore these mechanisms, we undertook multi-faceted analyses of whole lipedema tissue, ADSCs, and adipocytes, using omics platforms. Our integrated approach uncovered deranged metabolic and lipid profiles, and dysregulated signaling pathways driving key biological processes, such as adipogenesis, cell cycle, lipid metabolism, and cell proliferation and differentiation. The global molecular profiles we characterized in lipedema uniquely distinguish lipedema from normal fat and define it as a distinct disease tissue. Importantly, the findings of our multimodal study were coherent across distinct biological platforms and provide new insights that will both help to address the deficit in lipoedema animal models and provide vital clues into potential novel therapeutic approaches.

Lipids play important roles in physiological processes such as energy homeostasis, bioenergetics, apoptosis, signal transduction, and cell recognition. Defective lipid catabolic pathways are involved in the pathogenesis of several metabolic diseases [49]. Neutral lipids, such as triglycerides, are less likely to promote metabolic disorders compared to other lipids classes such as sphingolipids and GPLs [50, 51], which have been documented to be involved in several metabolic diseases [52]. An example of the distinct nature of lipedema adipose tissue is illustrated by our discovery of metabolic differences in lipedema relating to levels of fatty acids and their conjugates that included GPLs (more highly abundant in lipedema adipocytes than non-lipedema adipocytes) that are metabolites known to regulate cell-cycle events in cell growth, and to enhance cell size and proliferation [53, 54]; notably, GPL inhibition can reduce cell proliferation [55, 56]. Sphingolipids were the second most different class of lipid metabolites, in terms of metabolite levels, between lipedema and non-lipedema adipose tissues. Sphingolipids are among the lipid classes implicated in lipotoxicity, and can modulate signaling pathways involved in fibrosis, apoptosis, triglyceride synthesis, and glucose metabolism [57–59]. Recent studies have shown a role for sphingolipids in the development of metabolic disorders such as atherosclerosis, hepatic-steosis, cardiomyopathy, diabetes, and insulin resistance [60, 61]. Our finding that the adipocyte lipidomics profile is significantly altered in lipedema, particularly in relation to sphingolipid metabolites, suggests that a disrupted lipid profile in adipocytes could contribute to metabolic dysfunction and pathogenesis in lipedema.

The gene expression profiles defined in lipedema whole tissue and ADSCs highlighted potential molecular drivers of lipedema. We identified over 7820 significantly differentially expressed genes in RNA-seq data from lipedema adipose tissue and ADSCs (4391 in adipose tissue and 3429 in ADSCs). The top-ranking signaling pathways and GO-molecular functions identified related to cell cycle and mitotic spindle checkpoints. Detailed pathway analysis further implicated the involvement of genes from mitotic spindle checkpoint pathways. Validation of the eight most promising genes involved in mitotic spindle checkpoint regulation by qRT-PCR identified Bub1 as a potential key mediator of mitotic spindle checkpoint pathways in lipedema tissue and ADSCs. Of the candidates, Bub1 was chosen for further study due to the role it plays in proliferation in other diseases, as aberrant Bub1 expression has been shown to promote cell proliferation in several cancers [33, 43, 62], and higher Bub1 expression in lipedema tissue and ADSCs could disrupt G1/S checkpoint inhibition resulting in increased cell proliferation. Furthermore, cell-cycle analysis identified differences between the cell-cycle phases of ADSCs in lipedema (high proportion in S phase) and normal ADSCs (low proportion in S phase). The higher Bub1 expression in lipedema tissue and ADSCs may act to disrupt G1/S checkpoint inhibition resulting in increased cell proliferation. Mitosis also plays a role in balancing stem cells between the states of self-renewal and differentiation from progenitor to mature cells by regulating symmetric and asymmetric division [43, 63]; hence, Bub1 upregulation in lipedema may contribute to abrogation of stem cell homeostasis. Importantly, the Bub1 downstream target histone H2A, phosphorylation of which is considered important for cell proliferation, was more highly phosphorylated in lipedema ADSCs.

We therefore conducted further investigation of the role of Bub1, studying the effects of Bub1 inhibition on ADSC proliferation using 2OH-BNPP1 [64]. Excitingly, either 2OH-BNPP1 treatment or CRISPR/Cas9-mediated *Bub1* gene depletion can dramatically reduced lipedema ADSC proliferation. Treatment of lipedema ADSCs with 2OH-BNPP1 also profoundly inhibited histone H2A phosphorylation, further supporting a key role for Bub1 in enhanced ADSC proliferation and pathological adipogenesis in lipedema.

Taken together, our data—derived from multi-platform Omics interrogations across whole adipose tissue, adipocytes, and ADSCs derived from lipedema and control unaffected patients—have provided valuable insights into the mechanisms underlying lipedema. Using gene expression analysis and cell-based bioassays, we identified Bub1 as a novel therapeutic target in lipedema for which a small molecule inhibitor has been successfully tested in pre-clinical cancer models [65]. Our data indicate that Bub1 overexpression promotes ADSC hyperproliferation in lipedema and may therefore play a key role in lipedema disease onset and progression.

The findings of this study present, for the first time, a useful platform for pinpointing diagnostic biomarkers that may be predictive of lipedema disease progression. They may also help identify potential therapeutic targets for combating this chronic, incurable, and under-appreciated disease.

## Materials and methods

### Collection of human tissue and isolation of ADSCs

Adipose tissues were obtained by excision from the lower limbs of 24 non-obese female patients (14 with stage II–III lipedema (clinical criteria per Wold et al. [66]) and 10 non-lipedema healthy controls) who consented for inclusion in this study. Protocols were approved by the Human Research Ethics Committee, St Vincent’s Hospital, Melbourne (HREC-A 067/16 and HREC/16/SVHM/38) in accordance with the Declaration of Helsinki. ADSCs were isolated from adipose tissue from lipedema and non-lipedema control patients, using methods described by Zuk et al. [67] and as described in detail in Supplementary Methods.

### RNA-seq analyses

Adipose tissue and ADSC samples from LED and non-LED patients were used for global gene expression analyses by RNA-sequencing. An average of 78 million 100-base long reads from each adipose tissue and ADSC sample were mapped to the human reference genome (b37 decoy), and raw gene counts were obtained for each sample using STAR (v2.7.2c) as described in detail in Supplementary Methods. Downstream differential gene expression and gene-set analyses were then conducted in R (v3.3.0) using packages mainly including edgeR (v3.16.5) [68] and limma (v3.30.12) [69].

### Lipidomics and metabolomics analyses

ADSCs were cultured on 6-well culture dishes (*n* = 4) at 40,000 cells/cm^2^ in differentiation medium for 14 days. After 14 days, differentiated cells (adipocytes) were used for lipdomics and metabolomics analyses as described in detail in Supplementary Methods. Resulting lipidomic and metabolic profiling comparisons and PCA were undertaken using LCMS, and resulting untargeted data analyzed using IDEOM software [70]. Refer to Supplementary Methods for additional information.

### Time-lapse microscopy

Live imaging was performed over 72 h at 37 °C with 5% CO_2_ on an Operetta high-content screening system (PerkinElmer) using a 10× objective. Detail has been described in Supplementary Methods.

### Colony forming unit (CFU) assay

The CFU assay was used to assess ADSC self-renewal capacity. Individual colonies of >50 cells were counted using a microscope, dissolved with 0.5% SDS and absorbance measured at 539 nm (POLARstar Optima plate reader (BMG Labtek)). Detail has been described in Supplementary Methods.

### Cell-cycle assay

ADSC cell-cycle phase changes were monitored with FxCycle Cell Cycle Assay Kits Cat#F10348 (Invitrogen) as described in detail in Supplementary Methods. After 48–96 h incubation, cells were fixed with 4% PFA and stained with FxCycle Far Red stain (30 min). Samples were analyzed by flow cytometry (BD FACS ARIA Flow cytometer) using FlowJo software (Tree Star, Oregon, USA).

### Protein analysis by immunoblotting

ADSCs were cultured overnight (density 5000–8000 cells/cm^2^), lysed in RIPA buffer (Thermo Fisher Scientific, USA) and total cellular protein quantified (Pierce BCA Protein Assay kit (Thermo Fisher Scientific, USA)). Membranes were blocked (Nupage Blocking buffer, cat#927–40000, Li-COR, USA) and incubated with primary antibodies (Supplementary Table 6) as described in detail in Supplementary Methods.

### qPCR and gene expression analysis

Total RNA was extracted from ADSCs (RNeasy mini kit (Qiagen)), and 1 μg was transcribed to cDNA (gDNA Clear cDNA Synthesis Kit (Biorad)) and gene-specific primers (Supplementary Table 7) were used to perform PCR using the QuantStudio 6 (Applied Biosystem, Life Technologies). Detail has been described in Supplementary Methods.

### Flow cytometry

ADSCs were trypsinized, resuspended, and washed with FACS wash buffer. Cells were stained using antibodies against CD44 (1:20), CD105 (1:20), CD73 (1:20), or CD90 (1:20), or appropriate isotype controls (all antibodies from BD Pharmingen, Supplementary Table 6) according to the protocol as described in detail in Supplementary Methods. FACS analysis was performed on BD FACS ARIA Flow cytometer and FlowJo software (Tree Star, Oregon, USA).

### Cell proliferation assay

ADSCs were seeded in 96-well plates (3000 cells/well) with fresh culture media supplied 48 hourly. After 3 days in culture, cells were fixed (4% PFA), DAPI-nuclear stained and cell quantification performed using the Operetta high-content screening system (PerkinElmer, objectives ×10 magnification) as described in Supplementary Methods.

### Adipogenic differentiation assay and lipid droplet quantification

ADSCs were cultured on 96-, 48-, or 6-well culture dishes at 40,000 cells/cm^2^ in differentiation medium for 14 days. After 14 days, differentiated cells were fixed with 4% PFA (1 h), washed (PBS), and stained for lipid droplets with Bodipy (2 μg/mL in 150 mM NaCl) [71], before quantification was performed (Operetta High-Content Screening System, PerkinElmer, ×10 objective) as described in detail in Supplementary Methods.

### CRISPR/Cas9 to generate Bub1-knockdown ADSCs

We used lentiviral CRISPR/Cas9 particles (Invitrogen LentiArray CRISPR, Thermo Fisher Scientific, USA) to knock down Bub1 in ADSCs as described in detail in Supplementary Methods. Lentivector permanently expressing cas9 with a blasticidin-resistance gene, and lentivector expressing gRNA for the *Bub1* gene with a puromycin resistance gene, were used to infect ADSCs. *Bub1* gene knockdown was confirmed by qRT-PCR and western blotting.

### Immunohistochemistry of human tissue

Human tissue samples were fixed in neutral-buffered formalin and processed to paraffin wax. Five-millimeter sections were cut and mounted on Polysine slides and allowed to dry. Opal multiplex staining was performed according to the manufacturer’s (PerkinElmer) instructions, with minor modifications as described in detail in Supplementary Methods.

### Statistical analyses

Assays were performed in triplicate, each with ADSCs from at least three lipedema and non-lipedema patients. Data were reported as mean ± SEM. Statistical comparisons used unpaired two-tailed Student’s *t* test. Analyses were performed using GraphPad Prism 7.1 software. Comparison with *p* values < 0.05 were considered significantly different.

## Supplementary information

Supplementary Figure and Table Legends

Supplementary Methods

Supplementary Figure 1-1

Supplementary Figure 1-2

Supplementary Figure 1-3

Supplementary Figure 2

Supplementary Figure 3-1

Supplementary Figure 3-2

Supplementary Figure 4-1

Supplementary Figure 4-2

Supplementary Figure 4-3

Supplementary Figure 5-1

Supplementary Figure 5-2

Supplementary Figure 6

Supplementary Table 1

Supplementary Table 2

Supplementary Table 3

Supplementary Table 4

Supplementary Table 5

Supplementary Table 6

Supplementary Table 7
