# Supplementary material for: Key signaling networks are dysregulated in patients with the adipose tissue disorder, lipedema
Source: Int J Obes (Lond). 2021 Nov 11;46(3):502–14. doi: 10.1038/s41366-021-01002-1 (PMC8873020; doi:10.1038/s41366-021-01002-1)
Supplement: Supplementary file 1 — Supplementary Figure and Table Legends [file 41366_2021_1002_MOESM1_ESM.docx]

**Supplementary Figure and Table Legends**

**Supplementary Figure 1.** **(a)** Lipedema patients, stages I-IV. **(b)** Multi-dimensional scaling (plotMDS from limma) performed from the transcriptomic profiles of adipose tissue from 8 lipedema (LED18,19,25,26,27,28,31,32) and 4 non-lipedema (non-LED 3,8,10,12) patients indicates differences between lipedema and non-lipedema signatures. **(c)** Functional enrichment analysis of highly differentially regulated genes. Distribution of differentially expressed genes between lipedema and non-lipedema adipose tissues was annotated in Gene ontology (GO) and Cell signalling pathways. Top GO/cell signalling pathways were categorised according to logFold change and pValue significance (-log10(adj.P)). **(d)** Heatmaps of differentially expressed genes involved in Immune response, Lipid phosphorylation, Cell adhesion and Inflammatory response. **(e)** Heatmaps of differentially expressed genes involved in proliferation, cell adhesion, adipogenesis and estrogen response.

**Supplementary Figure 2.** **(a)** ADSCs were isolated from lipedema (LED) and non-lipedema (non-LED) adipose tissues and grown in monolayer culture. The cells (passage 0) were seeded to allow cell-cell contacts to form, and cells began to adopt characteristic spindle-shaped structures. Magnification x10, scale bar represents 50 mm. **(b)** Mesenchymal cell surface marker profiles of ADSCs were analysed by flow cytometry. Lipedema ADSCs were stained with CD105-APC/CD73-PE and CD90-APC/CD44-PE. The percentage of double positive CD105/CD73 (88.6%) and CD90/CD73 (89.2%) ADSCs was determined. **(c)** Mesenchymal cell surface marker profiles of ADSCs were analysed by RT-qPCR.

**Supplementary Figure 3.** **(a)** Non-supervised principal component analysis (PCA) plots from lipedema (n=4) and non-lipedema (n=4) adipocytes showed clustering of both groups. **(b)** Variable importance in project (VIP) score analysis to rank the lipids with differences in abundance between the lipedema and non-lipedema study groups. **(c)** Distribution of lipid molecules shows top classes were Glycerophospholipids (466 molecules), Sphingolipids (72 molecules), Fatty acids (72 molecules) and Glycerolipids (51 molecules). **(d)** Heatmaps showing fold-changes in abundance of lipids, sorted by lipid class, in adipocytes derived from LED patients compared to non-LED patients. Data were normalised by total signal before fold-changes were calculated.

**Supplementary Figure 4.** **(a)** PCA plot of 4 biological replicates from lipedema and non-lipedema adipocytes showing clear separation of two different classes of metabolites. **(b)** Distribution of the metabolites by metabolite class analysis shows top classes were amino acid metabolism (89 metabolites), Lipids: Fatty Acyls (87 metabolites), Lipids: Glycerophospholipids (84 metabolites), Carbohydrate metabolism (46 metabolites) and Lipid metabolism (20 metabolites). **(c)** Heatmap of top 50 metabolites which shows differences between lipedema and non-lipedema adipocytes. **(d)** Volcano plot showing highly differentially expressed metabolites. **(e)** VIP score analysis to rank the metabolites with differences in abundance between the lipedema and non-lipedema study groups

**Supplementary Figure 5.** **(a)** Multi-dimensional scaling was (plotMDS from limma) performed from the transcriptomic profiles of lipedema (LED) ADSCs from seven patients (LED21,23,25,27,28,32,34) and of non-lipedema (non-LED) ADSCs from three patients (non-LED10,12,14) indicating differences between lipedema ADSC and non-lipedema ADSC gene expression signatures. **(b)** Functional annotation of differentially expressed genes in lipedema versus non-lipedema ADSCs shows top gene ontology pathways involved. Genes ranked by LogFC*(-log10(adj.P)). **(c)** Heatmap of differentially expressed genes between lipedema and non-lipedema ADSCs was annotated in Gene ontology and molecular function pathways. Top differentially expressed pathways involving cell cycle and proliferation genes are shown in heatmap. **(d)** Cell cycle pathway showing differentially expressed genes (Red:Up-regulated, Blue:Down-regulated) involved in regulating different phases of the cell cycle (G1, S, G2 and M).

**Supplementary Figure 6**. **(a)** Phase contrast images of cell cycle assay. Cells were pre-treated with 2OH-BNPP1 (25 μM) or DMSO control for 24 h. After 24 h, cells were stained with Cell-Clock Cell Cycle Assay kit. Cells in G0/G1, G1/S, S and G2/M are stained yellow, yellow/green, green, and dark blue, respectively. **(b)** Cell cycle distribution was determined by ImageJ. Cell numbers in G0/G1, G1/S, S and G2/M phase are indicated (mean ± SE of three independent experiments).

**Supplementary Table Legends**

**Supplementary Table 1**. Characteristics of lipedema and non-lipedema control patients.

**Supplementary Table 2**. Differentially expressed genes in lipedema and non-lipedema adipose tissues. RNA-seq analysis of RNA from these tissues identified 4,391 differentially expressed genes (logFold change >1, adjusted p value <0.05). Gene symbols and entrez gene IDs are shown.

**Supplementary Table 3**. Comparison of lipids in lipedema versus non-lipedema adipocytes. More than 200 lipid species which were present at different levels in adipocytes from lipedema (n=4) compared to non-lipedema (n=4) patients are listed (p value <0.05). The ID and formulae of putative metabolites are also shown.

**Supplementary Table 4**. Comparison of metabolites in lipedema versus non-lipedema adipocytes. More than 150 metabolites are listed which exhibited significant differences (p value <0.05) in levels between adipocytes of lipedema (n=4) and non-lipedema (n=4) patients. The mass values, formulae and names of putative metabolites are shown.

**Supplementary Table 5**. Differentially expressed genes in lipedema versus non-lipedema ADSCs. RNA-seq analysis of RNA from these cells identified 3,429 differentially expressed genes (logFold change >1, adjusted p value <0.05). Gene symbols and entrez gene IDs are shown.

**Supplementary Table 6**. Antibodies used in this study.

**Supplementary Table 7**. Oligonucleotide primers used in this study. Sequences of forward and reverse primers are shown.
