# Supplementary material for: Key signaling networks are dysregulated in patients with the adipose tissue disorder, lipedema
Source: Int J Obes (Lond). 2021 Nov 11;46(3):502–14. doi: 10.1038/s41366-021-01002-1 (PMC8873020; doi:10.1038/s41366-021-01002-1)
Supplement: Supplementary file 2 — Supplementary Methods [file 41366_2021_1002_MOESM2_ESM.docx]

**Supplementary Methods**

**Isolation and expansion of ADSCs from human adipose tissue**

Adipose tissues were obtained by excision from the lower limbs of 24 BMI matched (26-36) female patients (14 with stage II-III lipedema (clinical criteria per Wold et al.^1^) and 10 non-lipedema healthy controls) undergoing elective debulking surgery who consented for inclusion in this study. Protocols were approved by the Human Research Ethics Committee, St Vincent’s Hospital, Melbourne (HREC-A 067/16 & HREC/16/SVHM/38) in accordance with the Declaration of Helsinki. ADSCs were isolated from adipose tissue (harvested as whole tissue to avoid trauma/stress effects of liposuction of tissue) from lipedema and non-lipedema control patients, using methods described by Zuk et al.^2^. Tissues (10 g) were washed (PBS), morselized, then dissociation with 1 mg/ml of collagenase type I (Worthington cat#LS004196) for 1 h at 37ºC with shaking speed 150 g. Collagenase enzyme was neutralized by adding 20 ml of growth medium with 10% FBS. The tissue suspension was spun at 300 g for 5 minutes to collect the SVF cells. Cells from the SVF pellet were filtered (100 μM filter) and seeded on culture plates (5,000-8,000 cells/cm^2^ in DMEM-HG, 10% FBS) in growth media (37ºC, 5% CO2), and ADSCs expanded and used at P3-5 for experiments.

**RNA-seq library preparation**

Adipose tissue and ADSC samples were used for global gene expression studies. RNA sample quality was analysed by Agilent 2100 Bioanalyser and Agilent RNA 6000 Nano kit (Agilent) to confirm RIN values were above 7, and the concentration of RNA was determined with Qubit High Sensitivity RNA kit (Thermo Fisher).  We then used 1 µg of total RNA for purification of polyadenylated RNA with oligo dT beads (New England BioLabs).  Polyadenylated RNA was used for RNA-seq library preparation with the KAPA RNA Hyperprep kit (Roche) according to the manufacturer’s guidelines.  Briefly, RNA was fragmented (approximate median length 200 nt) and converted to cDNA, followed by end-repair and A-tailing. Adapters compatible with Illumina sequencing were ligated to the cDNA. A concentration of 1.5 µM unique index sequences, also known as barcodes, were added to each library during the adapter ligation. These barcodes are used during data analysis to make a distinction from each sample. Next, we performed a post-ligation clean-up with 0.65X volume of AxyPrep Mag PCR Clean-up beads (Axygen) to remove excess adapters.  The barcoded libraries were amplified with 10 cycles of PCR and cleaned with a 1X volume of AxyPrep Mag PCR Clean-up beads.  Library sizes were confirmed with the Agilent 2100 Bioanalyser and Agilent High Sensitivity DNA kit and yields were confirmed with the Qubit dsDNA HS assay. Libraries were then diluted to generate 4 nM stocks.  Multiple libraries were then pooled together in equimolar ratios and sequenced in the same run on the Illumina NextSeq500 using a 2x150 cycle high output kit.

**Bioinformatics analysis and differential gene expression**

Raw sequencing data, averaging 19.5 million reads per sample, were quality checked using FastQC (version 0.11.9). Adapters were trimmed using cutadapt (version 2.10, with the following settings: minimum-length 18, error-rate 0.2, overlap=5). Reads were mapped against the human reference genome (b37 decoy) using the STAR spliced alignment algorithm (version 2.7.2c, with default parameters and chimSegmentMin 20)^3^ returning an average unique alignment rate of 90.1%. Raw gene counts were generated from STAR by supplying the option ‘quantMode *GeneCounts’*, which were then processed in R for testing of differential gene expression^4^. The raw gene counts were transformed into log2-CPM values using the cpm function with *log=TRUE* from edgeR. Genes with a CPM of larger than 1 in more than 4 adipose tissue samples or in more than 3 ADSC samples were kept. The rest of the genes were considered as lowly expressed genes and removed. Normalisation was done using the method of trimmed mean of M-values (TMM) as performed using the *calcNormFactors* function in edgeR (version 3.32.0)^4, 5^. Sample heteroscedascity was modelled and removed using the *voomWithQualityWeights* function from limma, and linear modelling was carried out using the *lmFit* and *contrasts.fit* functions, followed by empirical Bayes moderation using the *efit* function. Differentially expressed genes were then obtained by decideTests with significance defined using an adjusted p-value cutoff of less than 5%. Multi-dimensional scaling (MDS) of samples was performed and the results were plotted using the *plotMDS* function on the log-CPM values in limma^6^.

**Gene-set testing**

Gene set testing was done by applying the camera method ^7^ to the c5 ontology gene sets and Hallmark gene sets from Broad Institute’s MSigDB collection ^8^. Selected up-regulated gene-sets (with FDR <0.05) in LED were plotted in heatmaps.

**OPAL double or quad staining**

Human tissue samples were fixed in neutral-buffered formalin and processed to paraffin wax. Five mm sections were cut and mounted on Polysine slides and allowed to dry. Opal multiplex staining was performed according to the manufacturer’s (Perkin Elmer) instructions, with minor modifications. Briefly, after dewaxing and hydration, sections were antigen-retrieved in 10 mM citrate buffer, pH 6.0, for 15 minutes at 99^o^C, followed by cool-down for the same time. Endogenous peroxidase was quenched in 3% hydrogen peroxide for 15 min and sections blocked in TNB protein block (Perkin Elmer) for 30 min. The first primary antibody in the multiplex protocol was then applied for 60 min or overnight (at 4^o^C) as appropriate, followed by a HRP-conjugated secondary antibody at 1:100 or OPAL polymer secondary antibody (for CD34 only) for 30 min. OPAL fluorophore was then applied at 1:200 for 10 min in amplification diluent. For second and subsequent primaries in the multiplex procedure, sections were again retrieved/stripped in citrate buffer as above to remove the preceding antibodies, and subjected to the same sequence of steps, over multiple staining cycles. Throughout the protocol washing was performed with tris-buffered saline (TBS) and sections shielded from light exposure as appropriate during incubations. Isotype antibody controls were performed to confirm specificity of staining, and antibody sequence optimised for balanced staining and imaging. At completion of final cycle, sections were mounted in Prolong Gold anti-fade reagent and allowed to dry prior to imaging. For CD34 and CD29 (both Abcam) double staining**,** CD34 was applied at 1:30,000 and detected with Opal 650, and CD29 subsequently at 1:100 with detection using Opal 520. Sections were imaged with an Olympus BX61 microscope with DP71 camera, using appropriate filters and a 20x objective. For perilipin, CD29, CD31 and CD34 quad multiplexing (in that order), perilipin (Cell Signaling) was applied at 1:3,200 and detected with Opal 620, CD29 at 1:150 with Opal 570 detection, CD31 (Invitrogen) at 1:20 with Opal 520 detection, and CD34 at 1:30,000 with Opal 650 detection. Imaging was performed using a Nikon A1R confocal microscope (Biological Optical Microscopy Platform, University of Melbourne*)* using the spectral detector with 6 nm resolution to separate the four colours. Images were acquired using 3 lasers (488, 561 and 635 nm), 40x oil objective without zoom, 1024x1024 resolution, and 3x3 tiled stitching. Sections stained with single fluorophores were used to define spectra and ensure adequate spectral separation in the images obtained from the multiplexed sections.

**Flow cytometry**

We used flow cytometry to validate ADSC-expressed markers. To achieve a single cell suspension, cells were trypsinized, resuspended and washed with FACS wash buffer. Cells were stained using antibodies against CD44 (1:20), CD105 (1:20), CD73 (1:20) or CD90 (1:20), or appropriate isotype controls (all antibodies from BD Pharmingen, Supp. Table 6). Cells were incubated with a saturated antibody solution for 30 min, then twice washed in cold FACS buffer, then resuspended in 150 ml of cold FACS buffer, and kept on ice until acquisition. FACS analysis was performed on BD FACS ARIA Flow cytometer and FlowJo software (Tree Star, Oregon, USA). Cells were acquired and gated by forward scatter (FSC) and side scatter (SSC) to exclude debris and cell aggregates. To calculate the percentage of cells positive for each of the selected markers, a maximum of 0.5% false positive gate was set using the isotype control.

**Protein analysis by immunoblotting**

ADSCs were cultured overnight (density 5,000-8,000 cells/cm^2^), lysed in RIPA buffer (Thermo Fisher Scientific, USA) and total cellular protein quantified (Pierce BCA Protein Assay kit, Thermo Fisher Scientific, USA). Total cellular protein (5-10 µg) was loaded on Nupage gel (4-12% Bis-Tris cat#NP0336, Invitrogen, USA), electrophoresed and transferred to iblot 2 PVDF membrane (cat#IB24002, Invitrogen, USA). Membranes were blocked (Nupage Blocking buffer, cat#927-40000, Li-COR, USA) and incubated with primary antibodies (Supp. Table 6) overnight (4ºC), then with secondary antibodies labelled with infrared dyes (IRDye 800CW and 680RD, 1:20,000 ratio in Nupage blocking buffer) before quantification (Odyssey Imaging System (LI-COR)). GAPDH antibody was used to normalize samples. We used housekeeping protein normalization protocol (licor.com/HKP-validation, Li-COR, USA) by employing Image Studio Software (licor.com/islite). Lane normalization factors were determined from each GAPDH band and each target protein was then normalized with the lane normalization factor. Normalized band intensities were presented in graphs as arbitrary units (a.u.) for relative comparison of samples.

**Cell Cycle Assay**

ADSC cell cycle phase changes were monitored with FxCycle Cell Cycle Assay Kits Cat#F10348 (Invitrogen). ADSCs were seeded onto 24-well plates (2x10^4^ cells/well) then incubated for 24 h, at 37ºC. Cells were synchronized with serum-starved media (2% FBS, DMEM) overnight and then incubated in full growth media (10% FBS, DMEM). After 48-96 h incubation, cells were fixed with 4% PFA and stained with FxCycle Far Red stain (30 min). Samples were analysed by flow cytometry (BD FACS ARIA Flow cytometer) using FlowJo software (Tree Star, Oregon, USA). Changes in the cell cycle phases of ADSCs after treatment with Bub1 inhibitor BNPP-2OH were monitored by using the Cell-Clock ^TM^ Cell Cycle Assay kit (Biocolor Life Science Assays, Carrickfergus, UK). LED and non-LED ADSCs were seeded in 24 well plates (2x10^4^ cells/well) and incubated for 24 hrs at 37℃. Cells were then synchronized with serum starve media (DMEM with 2%FBS) overnight. After 24 h of BNPP-2OH treatment of ADSCs, Cell-Clock dye (150 µl) was added and incubated for 1 h at 37℃. Cells were washed twice gently with pre-warmed DMEM. Stained cells were imaged using Olympus BX61 microscope. Cells in G0/G1, G1/S, S and G2/M are stained yellow, yellow/green, green, and dark blue, respectively. Color pixels from at least 6 photomicrographs for each sample were quantified and averaged using ImageJ software.

**Time lapse microscopy**

Live imaging was performed over 72 h at 37^o^C with 5% CO_2_ on an Operetta high-content screening system (PerkinElmer) using a 10x objective. Nine fields of view were captured using brightfield. To measure cell growth over time the phonologic machine learning module of Harmony was trained to segment the cell coverage area from the background. The area covered by cells was logged. Data were analysed by standardising the area covered over time against the area of cells at the first time point for each well.

**Colony forming unit (CFU) assay**

The CFU assay was used to assess ADSC self-renewal capacity. Single cell suspensions (P1) were plated in six-well plates (1x10^4^ cells/well) in growth medium (DMEM with 10% FBS) and media was changed after every 2-3 days. On day 14, fixed cells (4% PFA, 10 min) were stained with 0.5% crystal violet. Individual colonies of >50 cells were counted using a microscope, dissolved with 0.5% SDS and absorbance measured at 539 nm (POLARstar Optima plate reader (BMG Labtek)).

**Gene expression analysis**

Total RNA was extracted from ADSCs (RNeasy mini kit (Qiagen)), and 1 μg was transcribed to cDNA (gDNA Clear cDNA Synthesis Kit (Biorad)), and qPCR performed using cDNA equivalent to 10-20 ng RNA and a Taqman PCR master mix or SYBR green master mix (Biorad) and primers (Supp. Table 7). Samples were analysed in duplicate and PCR performed using the QuantStudio 6 (Applied Biosystem, Life Technologies).

**Cell proliferation assay**

ADSCs were seeded in 96-well plates (3,000 cells/well) with fresh culture media supplied 48 hourly. After 3 days in culture, cells were fixed (4% PFA), DAPI-nuclear stained and cell quantification performed using the Operetta high-content screening system (PerkinElmer, objectives x10 magnification). Nine fields of view per well were imaged and positive nuclei were counted using the “find nuclei” function in Harmony (v4.8). In some experiments, proliferation was measured (CellTiterGlo proliferation assay (Promega)) as per manufacturer’s protocols. Luminescence was measured by a POLARstar Optima plate reader (BMG Labtek). In some experiments, ADSCs were treated with Bub1 inhibitor BNPP-2OH (Cat#HY-102081, MedChemExpress, USA) at 25 µM.

**Adipogenic differentiation assay and lipid droplet quantification**

ADSCs were cultured on 96-, 48- or 6-well culture dishes at 40,000 cells/cm^2^ in differentiation medium (DMEM with 10% FBS, 1 μM dexamethasone, 500 μM 3-isobutyl-l-methyl-xanthine, 60 μM indomethacin, 5 μg/ml insulin (all from Sigma)) for 14 days. The medium was replenished every 3-4 days. After 14 days, differentiated cells were fixed with 4% PFA (1 h), washed (PBS), and stained for lipid droplets with Bodipy (2 μg/ml in 150 mM NaCl)^9^, before quantification was performed (Operetta high-content screening system, PerkinElmer, x10 objective). Quantification of lipid droplets was carried out in Harmony v4.8 (PerkinElmer) by first capturing images from 80% of the well and then analysig them as follows. Nuclei were segmented and counted using the “find nuclei” command. Bodipy droplets were then extracted and measured using the “find spots” command. Fat droplet- positive cells were classified as any extracted nuclei that had significant bodipy signals surrounding them.

**CRISPR/Cas9 to generate Bub1 knockdown ADSCs**

We used lentiviral CRISPR/Cas9 particles (Invitrogen LentiArray CRISPR, Thermo Fisher Scientific, USA) to knock down Bub1 in ADSCs. We plated 5,000 cells/cm^2^ (50-60% confluence) and used blasticidin (2.5 μg/ml) and puromycin (0.3 μg/ml) for *Cas9* and *Bub1* gene selection. Multiplicity of infection (MOI) was determined by using LentiArray CRISPR Positive lentivirus (Cat#A32060) particles to allow maximum knockdown efficiency. Lentivector permanently expressing cas9 with a blasticidin-resistance gene, and lentivector expressing gRNA for the *Bub1* gene with a puromycin resistance gene, were used to infect ADSCs (MOI ratio of 5-10 lentivirus particles). To minimize toxicity from virus, virus was aspirated and fresh complete growth media added 24 h post-transduction and cells incubated at 37℃ in humidified 5% CO_2_ for 2-5 days. We used positive control lentivector with gRNA for the *HPRT* gene and negative controls with scrambled gRNA. *Bub1* gene knockdown was confirmed by qRT-PCR and Western blotting.

**Lipidomics and Metabolomics**

***Materials and Instrumentation***

LCMS grade acetonitrile was from Burdick and Jackson (Muskegon, MI, USA). Ammonium carbonate was purchased from Sigma Aldrich (St. Louis, Missouri, United States). LCMS grade formic acid (Optima) was from Fisher Chemical (part of Thermo Fisher Scientific, Waltham, Massachusetts, USA). MilliQ water was from Millipore water purification system (Merck KGaA, Darmstadt, Germany).

LCMS data was acquired on Q-Exactive Orbitrap mass spectrometer (Thermo Scientific, Waltham, Massachusetts, USA) coupled with high-performance liquid chromatography (HPLC) system Dionex Ultimate® 3000 RS (Thermo Scientific, Waltham, Massachusetts, USA). The samples were analyzed as a single batch to reduce the batch-to-batch variation and randomized to account for LCMS system drift over time.

***Lipidomics LC-MS data acquisition***

Chromatographic separation was performed on Agilent Zorbax C8 (1.8 µm, 2.1 x 100 mm, Agilent Technologies) equipped with a guard column (C8, 2 x 2 mm, Phenomenex). The mobile phase (A) was 40 % isopropanol, 8 mM ammonium formate, 2 mM formic acid and (B) 98% isopropanol, 8 mM ammonium formate, 2 mM formic acid, and needle wash solution was 50% isopropanol. The gradient program started at 0% B and was increased stepwise to 20% B over 1.5 min, to 28% B over 5.5 min, to 35% B over 1 min, to 65% B over 16 min and to 100% B over 1 min. Wash at 100% B was continued for 2 min before decreasing to 0% B over the next 2 min followed by equilibration at 0% B for 1 min. The flow rate was 0.2 ml/min and column compartment temperature 40ºC. The total run time was 30 min with injection volume of 10 µl. The mass spectrometer operated in full scan mode with positive and negative polarity switching at 70k resolution at 200 m/z with detection range of 140 to 1300 m/z, AGC target 1e6 ions, maximum injection time 50 ms. Electro-spray ionization source (HESI) was set to 3.5 kV voltage for positive mode and 3.5 kV for negative mode, sheath gas was set to 34, aux gas to 13 and sweep gas to 1 arbitrary units, capillary temperature 250°C, probe heater temperature 190°C.

***Metabolomics LC-MS data acquisition***

Chromatographic separation was performed on ZIC-pHILIC column (5 µm, 4.6 × 150 mm, SeQuant®, Merck) equipped with a guard (ZIC-pHILIC). The mobile phase (A) was 20 mM ammonium carbonate and (B) acetonitrile, needle wash solution was 50% isopropanol. The gradient program started at 80% B and was decreased to 50% B over 15 min, then to 5% B until 18 min, kept at 5% B until 21 min, returned to 80% B until 24 min and equilibrated at 80% B until 32 min. The flow rate was 0.3 ml/min and column compartment temperature 25ºC. The total run time was 32 min with an injection volume of 10 µl. The mass spectrometer operated in full scan mode with positive and negative polarity switching at 35k resolution at 200 m/z with detection range of 85 to 1275 m/z, AGC target 1e6 ions, maximum injection time 50 ms. Electro-spray ionization source (HESI) was set to 4.0 kV voltage for positive and negative mode, sheath gas was set to 50, aux gas to 20 and sweep gas to 2 arbitrary units, capillary temperature 300°C, probe heater temperature 120°C.

***Sample extraction***

Cell pellets were suspended in 1 ml chilled PBS and centrifuged at 1000 g for 5 min at 4ºC. The supernatant was discarded and the remaining solid was resuspended in 250 µl of chilled extraction solvent CHCl_3_/MeOH/water ((1 : 3 : 1, v/v) containing internal standards CAPS, CHAPS, PIPES and TRIS at 1 µM concentration). Blank sample containing water instead of cell pellet was extracted together. After three freeze-thaw cycles were performed the samples were mixed thoroughly for 30 min at 4°C. The samples were centrifuged at top speed for 10 min at 4°C. 100 µl of supernatant was transferred to the vials for metabolomics analysis and analysed on the same day. Another 100 µl was transferred to Eppendorf tubes for lipidomics analysis. The solvent was evaporated using a centrifugal evaporator at 55ºC for 50 min. Dried extracts were frozen at -80ºC until LCMS analysis was performed. On the day of analysis the samples were dissolved in 80 µl of BuOH/MeOH/water (4.5 : 4.5 : 1, v/v). The samples were shaken for 30 min at room temperature and kept in a sonicator bath for 1 h keeping the temperature below 25ºC. The samples were centrifuged at 14,800 g for 10 min at 20 ºC and 70 µl transferred to LCMS vials.

***Data processing using IDEOM***

The acquired LCMS data were processed in untargeted fashion using open source software IDEOM^10^ (http:// mzmatch.sourceforge.net/ideom.php), which initially used ProteoWizard to convert raw LC-MS files to *.mzXML* format and XCMS to pick peaks to convert to *.peakML* files. Mzmatch.R was subsequently used for the alignment of samples and the filtering of peaks using minimum detectable intensity of 100,000, relative standard deviation (RSD) of < 0.5 (reproducibility), and peak shape (codadw) of > 0.8. Mzmatch was also used to retrieve missing peaks and annotation of related peaks. Default IDEOM parameters were used to eliminate unwanted noise and artefact peaks. Loss or gain of a proton was corrected in negative and positive ESI mode, respectively, followed by putative identification of metabolites by accurate mass within 3 ppm mass error searching against the Kyoto Encyclopedia of Genes and Genomes (KEGG), MetaCyc, and LIPIDMAPS databases and others. For metabolomics, to reduce the number of false positive identifications, retention time error was calculated for each putative ID using IDOEM build-in retention time model which uses actual retention time data of authentic standards. ID’s of most significantly changed lipid species were confirmed using MSMS spectral data.

MSMS Statistical analysis on the data processed with IDEOM was performed using web application <http://www.metaboanalyst.ca>. For statistical analysis, data was normalised by sum, log transformed and auto scaled.

**References**

1. Wold LE, Hines EA, Jr., Allen EV. Lipedema of the legs; a syndrome characterized by fat legs and edema. *Ann Intern Med* 1951; **34**(5)**:** 1243-50.

2. Zuk PA, Zhu M, Mizuno H, Huang J, Futrell JW, Katz AJ *et al.* Multilineage cells from human adipose tissue: implications for cell-based therapies. *Tissue Eng* 2001; **7**(2)**:** 211-28.

3. Dobin A, Davis CA, Schlesinger F, Drenkow J, Zaleski C, Jha S *et al.* STAR: ultrafast universal RNA-seq aligner. *Bioinformatics* 2013; **29**(1)**:** 15-21.

4. Robinson MD, McCarthy DJ, Smyth GK. edgeR: a Bioconductor package for differential expression analysis of digital gene expression data. *Bioinformatics* 2010; **26**(1)**:** 139-40.

5. Lun AT, Chen Y, Smyth GK. It's DE-licious: A Recipe for Differential Expression Analyses of RNA-seq Experiments Using Quasi-Likelihood Methods in edgeR. *Methods Mol Biol* 2016; **1418:** 391-416.

6. Su S, Law CW, Ah-Cann C, Asselin-Labat ML, Blewitt ME, Ritchie ME. Glimma: interactive graphics for gene expression analysis. *Bioinformatics* 2017; **33**(13)**:** 2050-2052.

7. Wu D, Smyth GK. Camera: a competitive gene set test accounting for inter-gene correlation. *Nucleic Acids Res* 2012; **40**(17)**:** e133.

8. Subramanian A, Tamayo P, Mootha VK, Mukherjee S, Ebert BL, Gillette MA *et al.* Gene set enrichment analysis: a knowledge-based approach for interpreting genome-wide expression profiles. *Proc Natl Acad Sci U S A* 2005; **102**(43)**:** 15545-50.

9. Gocze PM, Freeman DA. Factors underlying the variability of lipid droplet fluorescence in MA-10 Leydig tumor cells. *Cytometry* 1994; **17**(2)**:** 151-8.

10. Creek DJ, Jankevics A, Burgess KE, Breitling R, Barrett MP. IDEOM: an Excel interface for analysis of LC-MS-based metabolomics data. *Bioinformatics* 2012; **28**(7)**:** 1048-9.
