# Supplementary figures and images for: Key signaling networks are dysregulated in patients with the adipose tissue disorder, lipedema
Source: Int J Obes (Lond). 2021 Nov 11;46(3):502–14. doi: 10.1038/s41366-021-01002-1 (PMC8873020; doi:10.1038/s41366-021-01002-1)

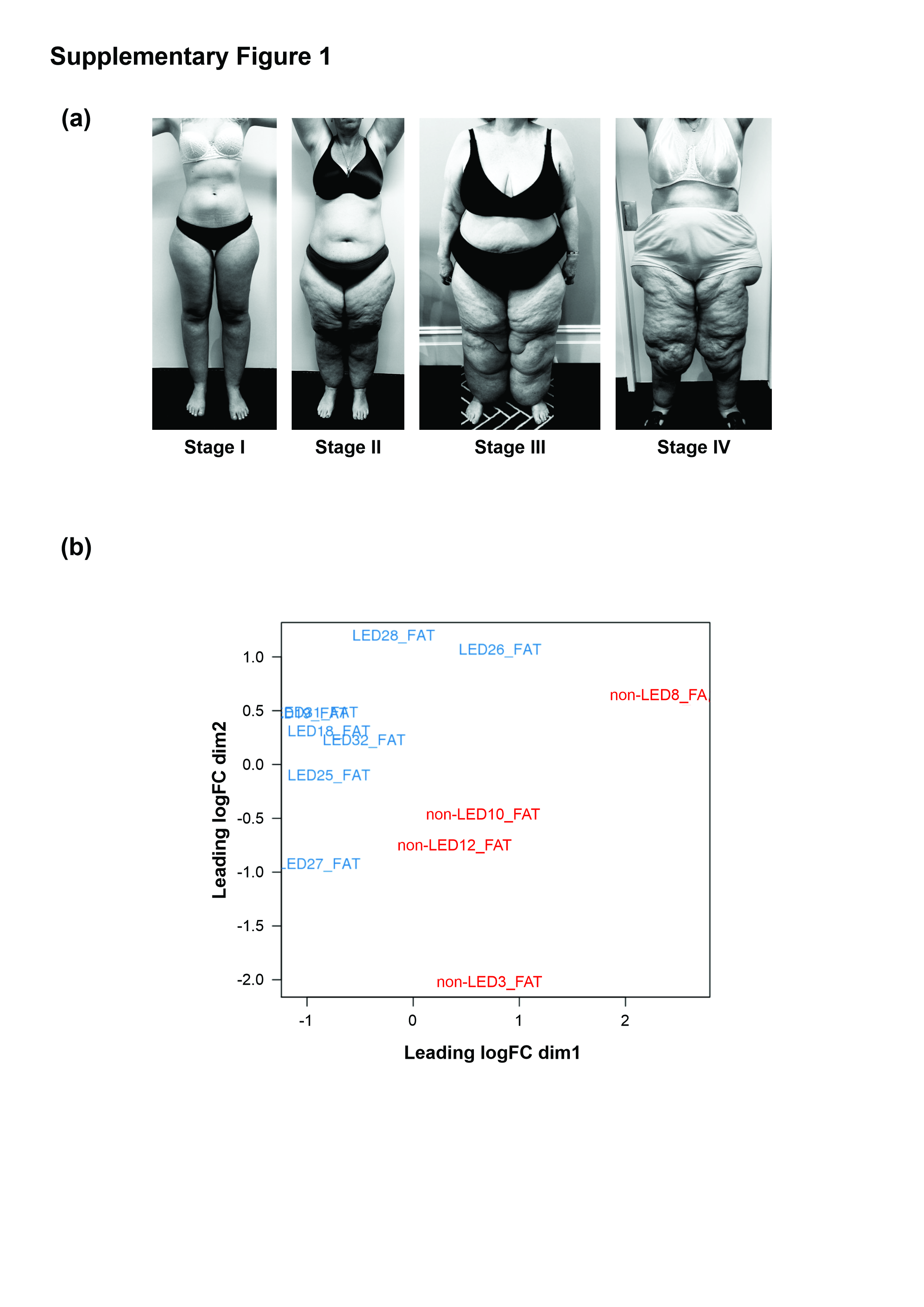

Supplement: Supplementary file 3 — Supplementary Figure 1-1 [file 41366_2021_1002_MOESM3_ESM.tif]

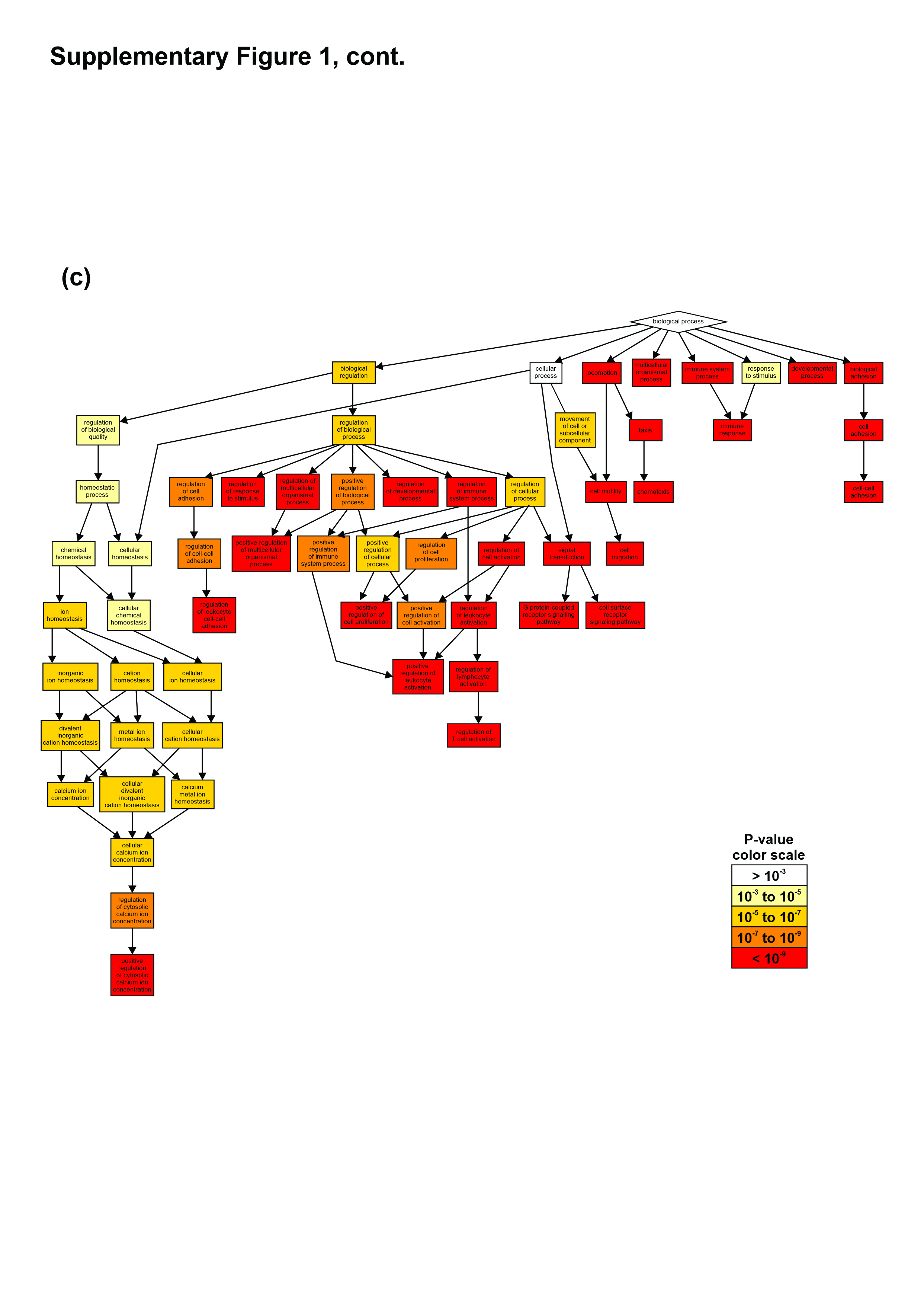

Supplement: Supplementary file 4 — Supplementary Figure 1-2 [file 41366_2021_1002_MOESM4_ESM.tif]

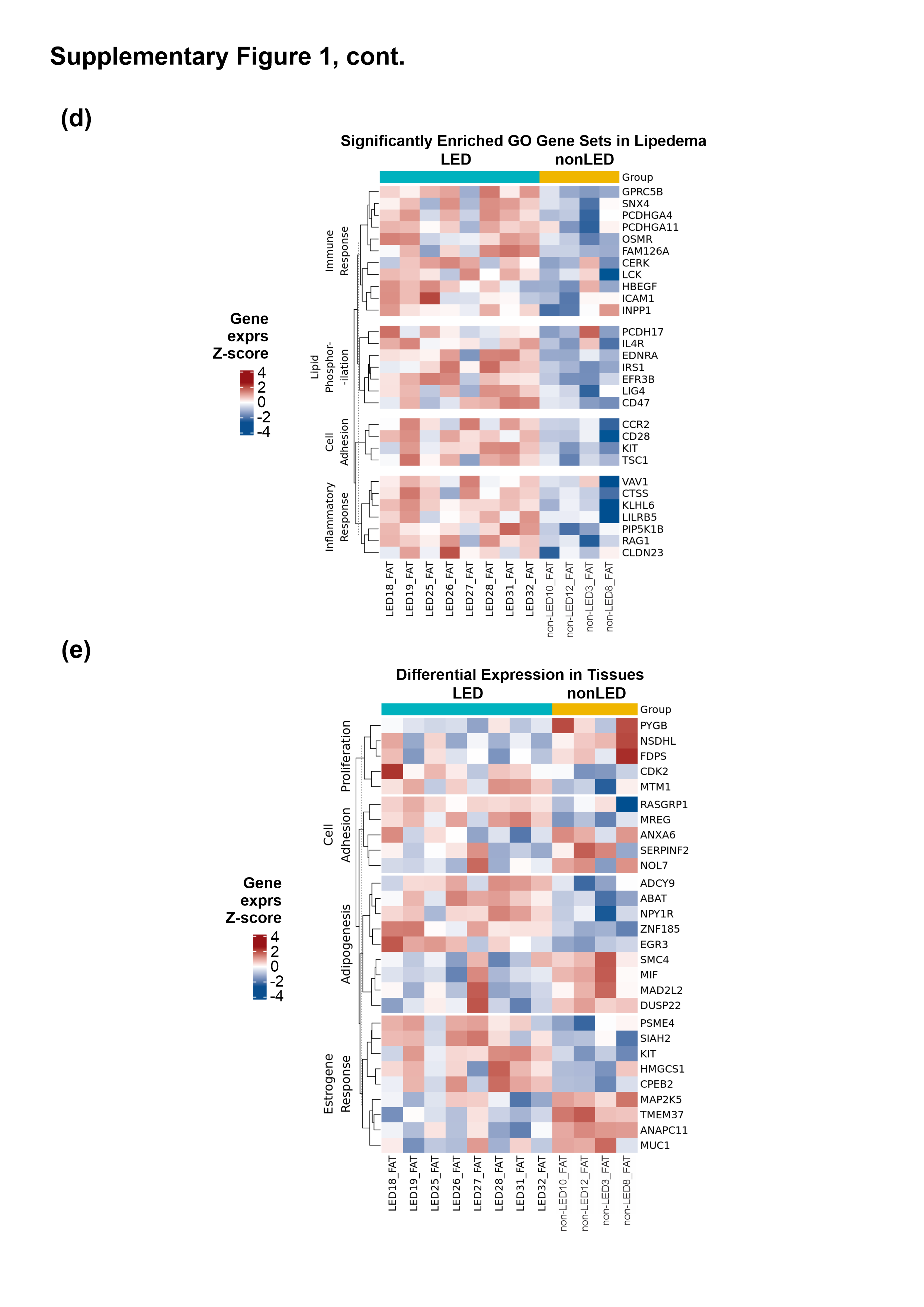

Supplement: Supplementary file 5 — Supplementary Figure 1-3 [file 41366_2021_1002_MOESM5_ESM.tif]

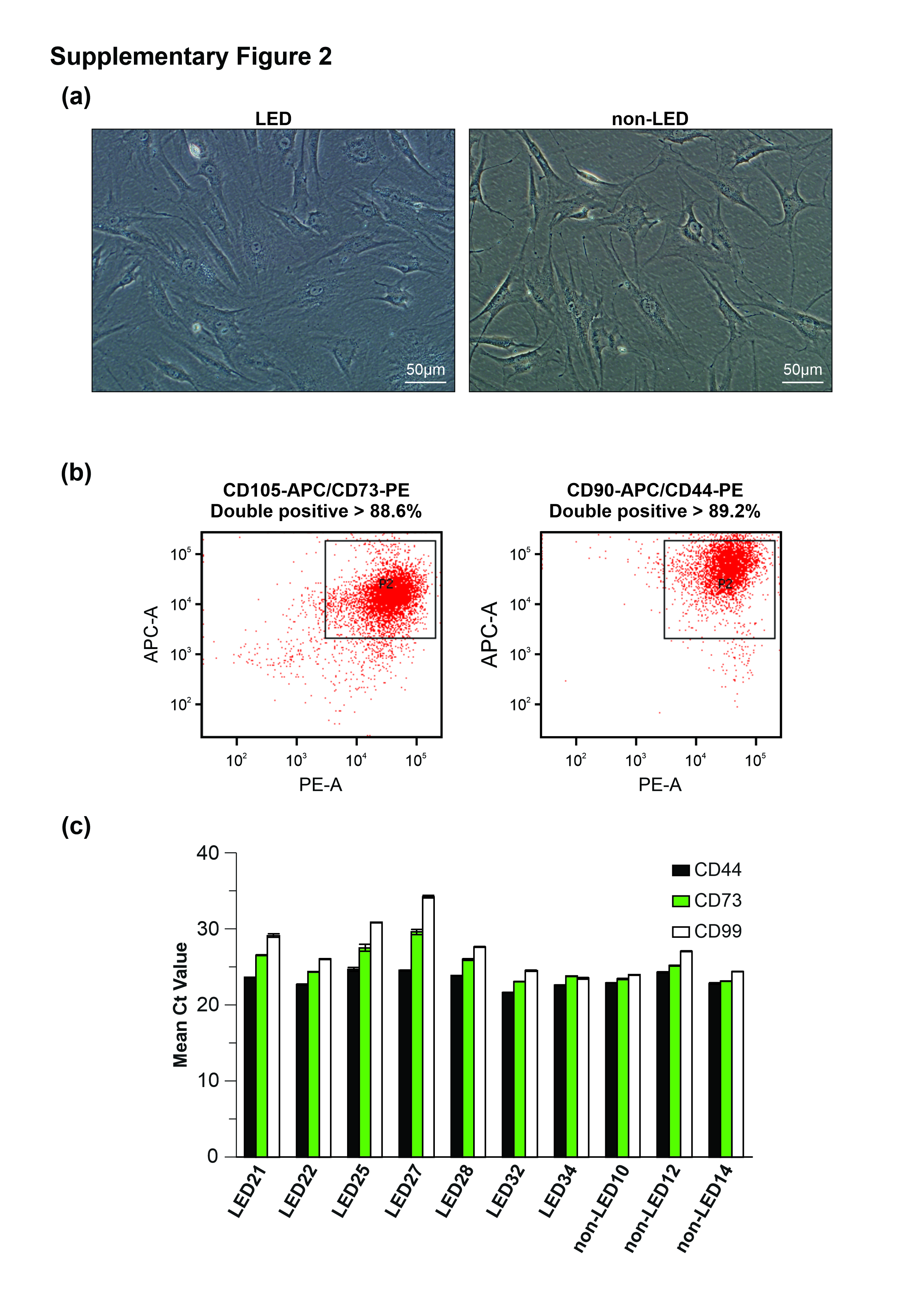

Supplement: Supplementary file 6 — Supplementary Figure 2 [file 41366_2021_1002_MOESM6_ESM.tif]

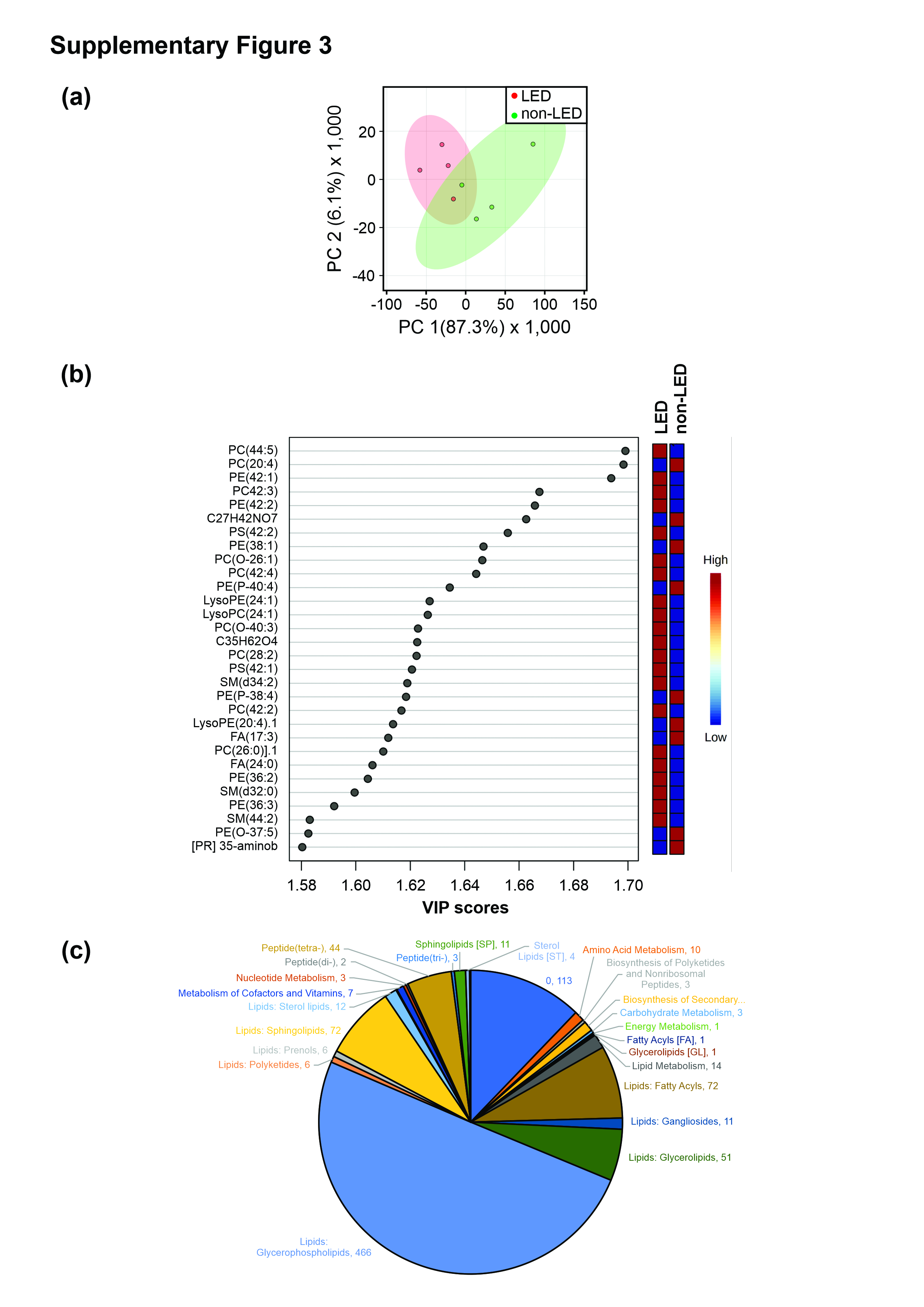

Supplement: Supplementary file 7 — Supplementary Figure 3-1 [file 41366_2021_1002_MOESM7_ESM.tif]

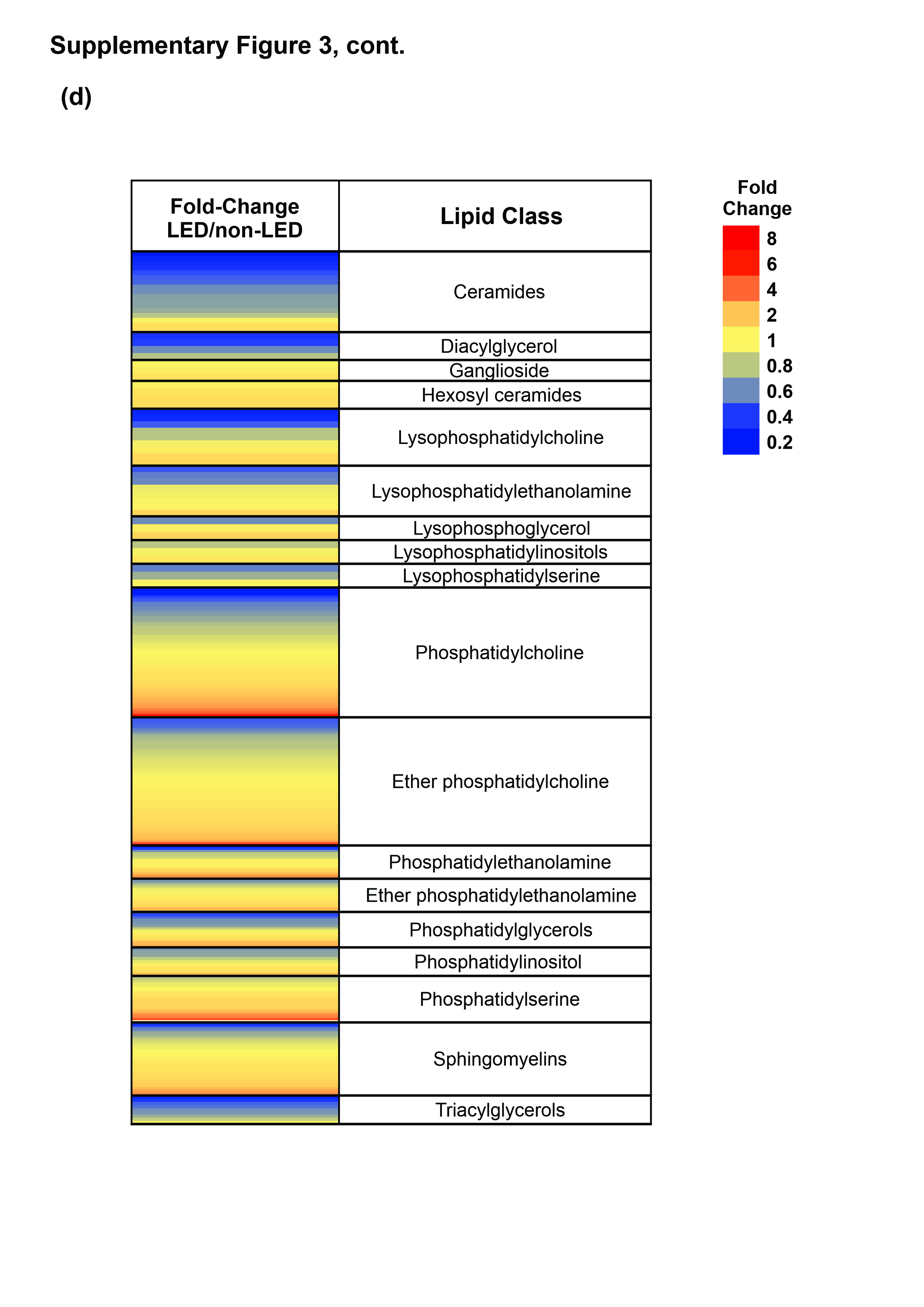

Supplement: Supplementary file 8 — Supplementary Figure 3-2 [file 41366_2021_1002_MOESM8_ESM.tif]

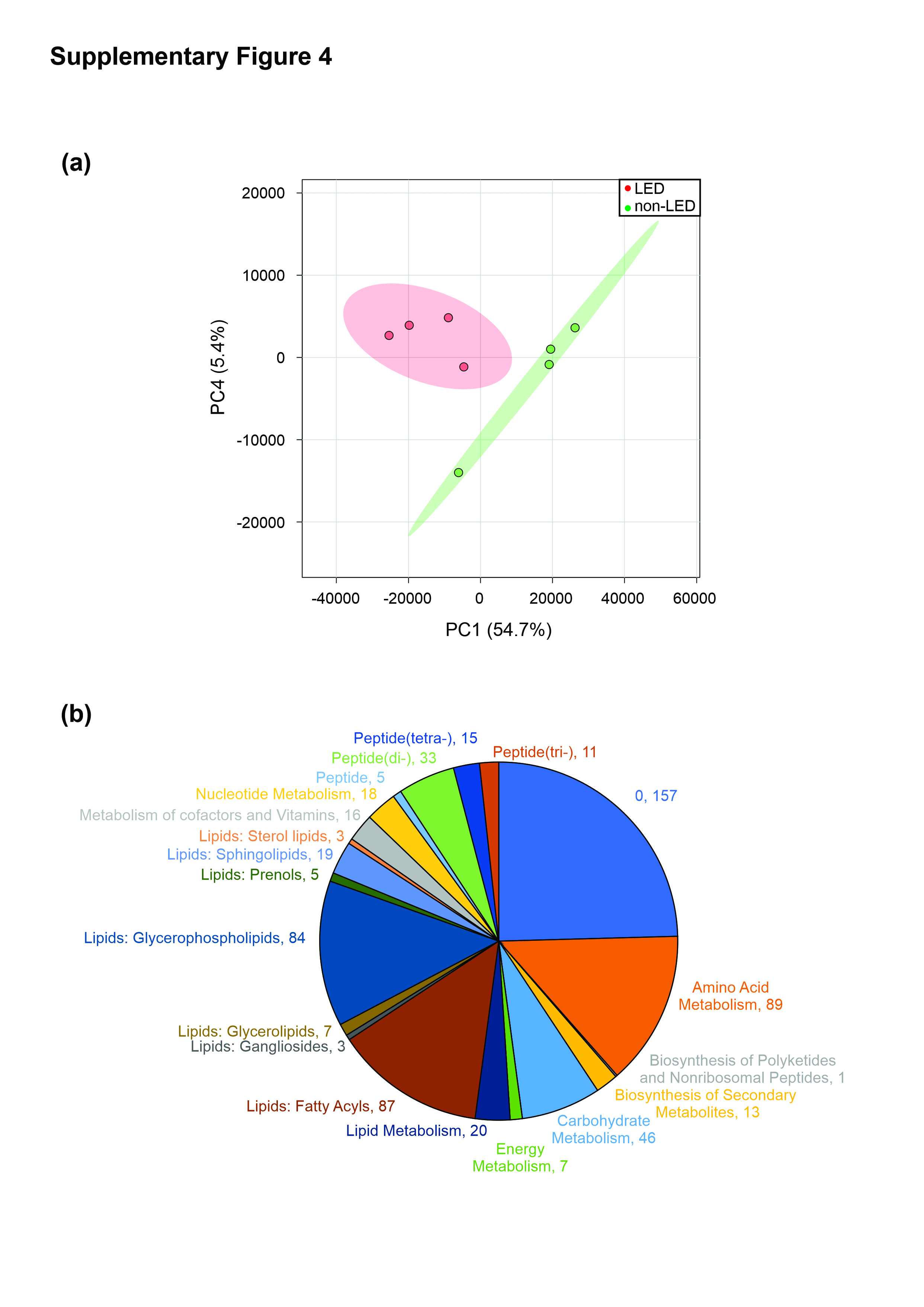

Supplement: Supplementary file 9 — Supplementary Figure 4-1 [file 41366_2021_1002_MOESM9_ESM.tif]

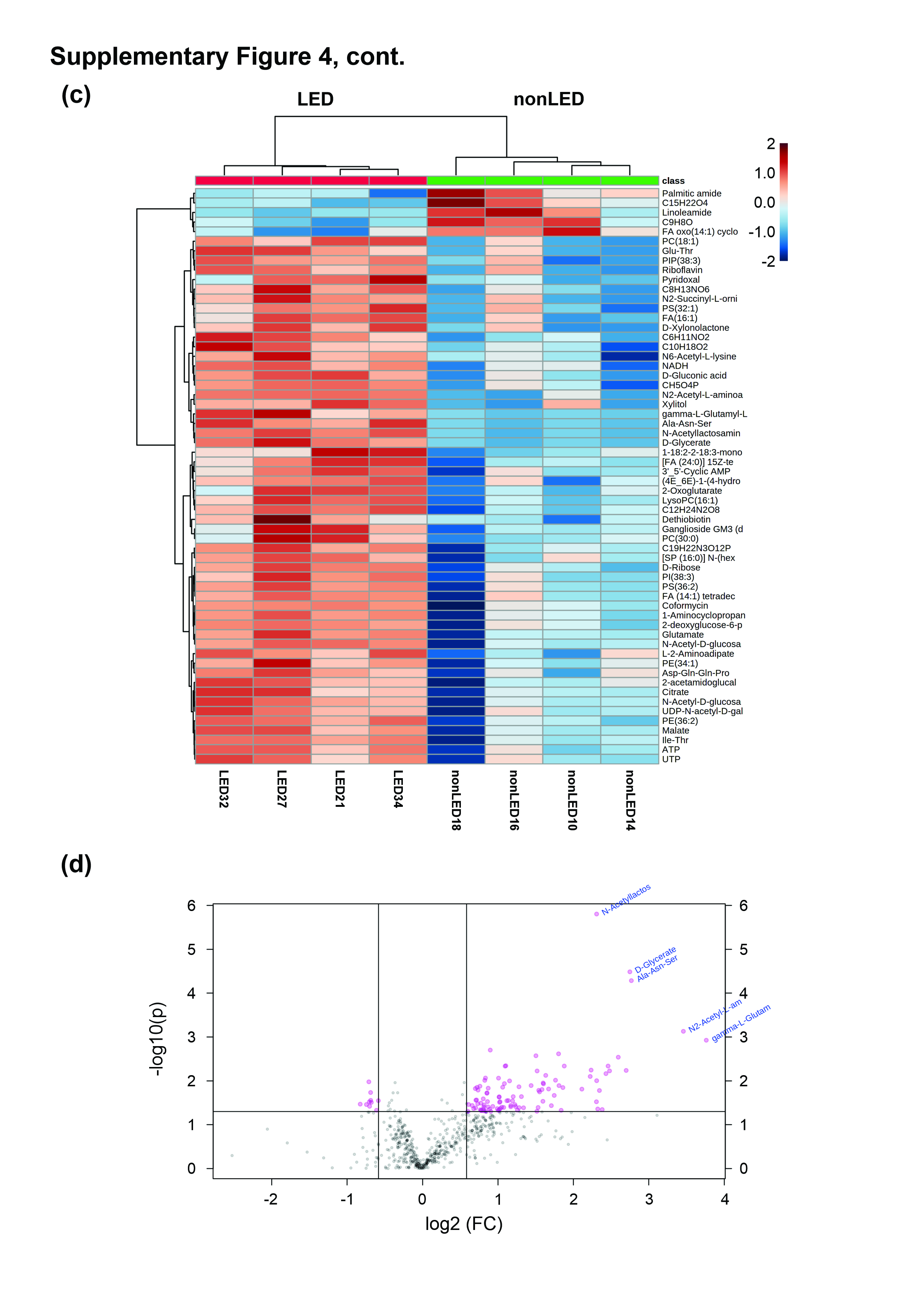

Supplement: Supplementary file 10 — Supplementary Figure 4-2 [file 41366_2021_1002_MOESM10_ESM.tif]

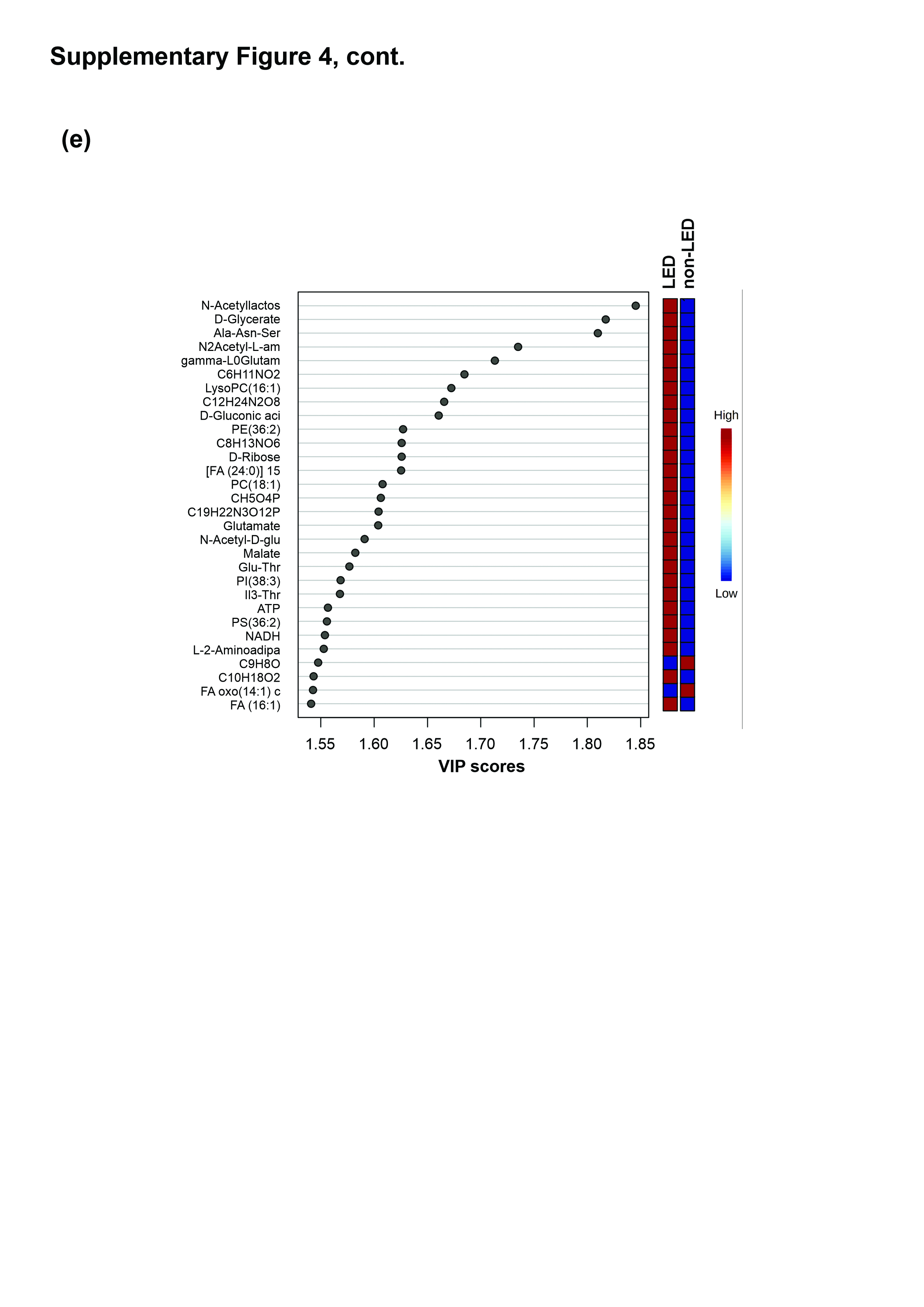

Supplement: Supplementary file 11 — Supplementary Figure 4-3 [file 41366_2021_1002_MOESM11_ESM.tif]

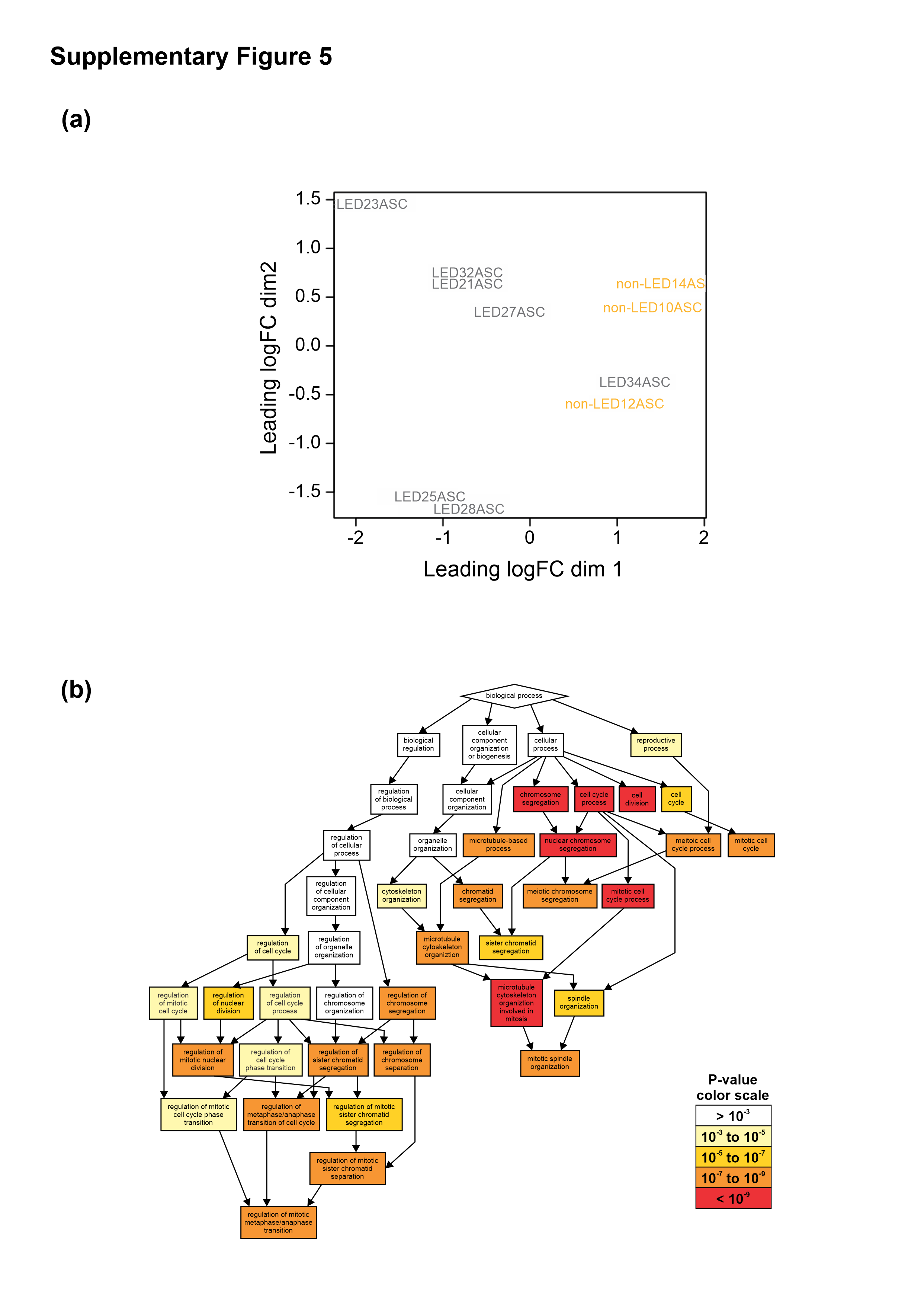

Supplement: Supplementary file 12 — Supplementary Figure 5-1 [file 41366_2021_1002_MOESM12_ESM.tif]

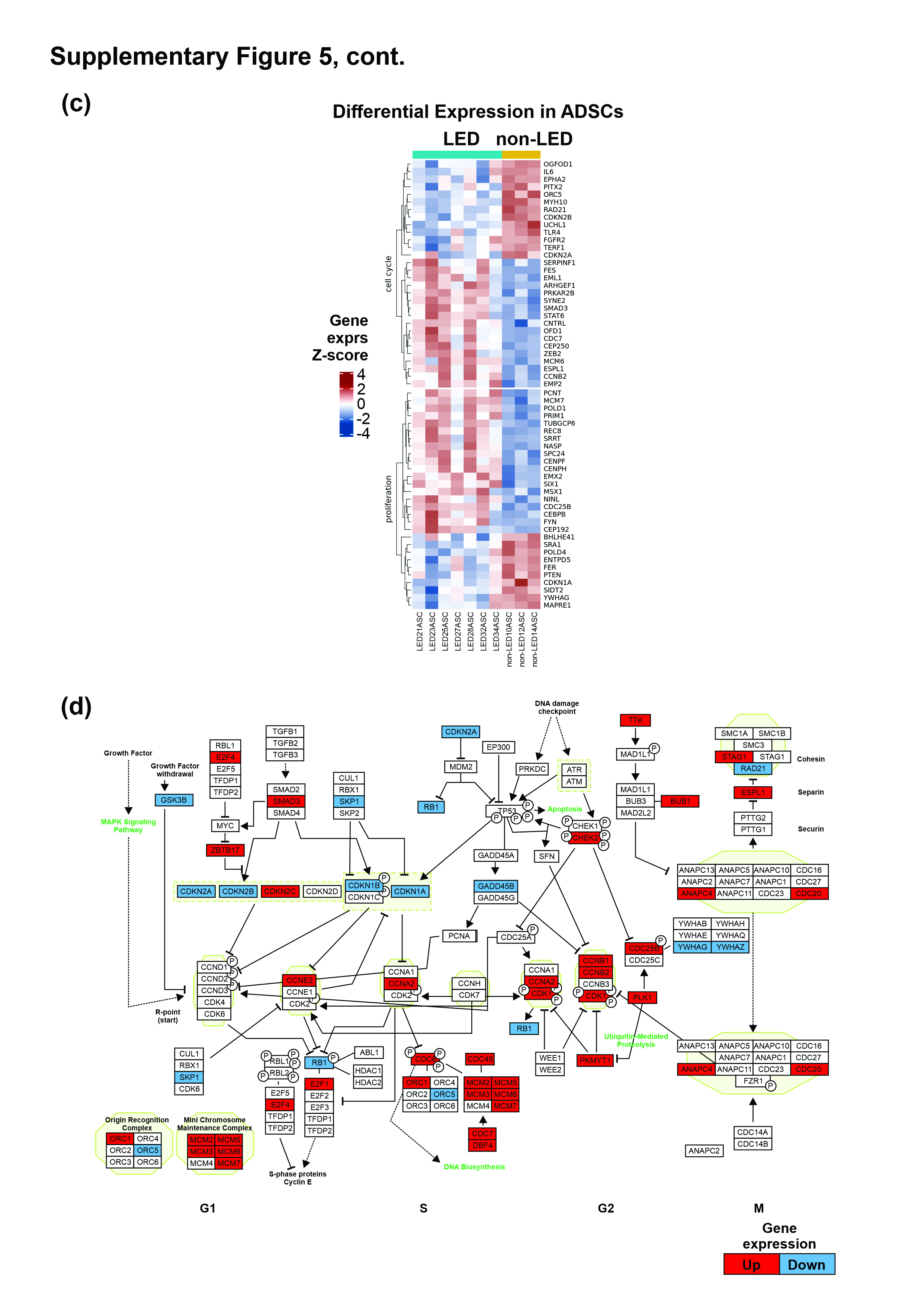

Supplement: Supplementary file 13 — Supplementary Figure 5-2 [file 41366_2021_1002_MOESM13_ESM.tif]

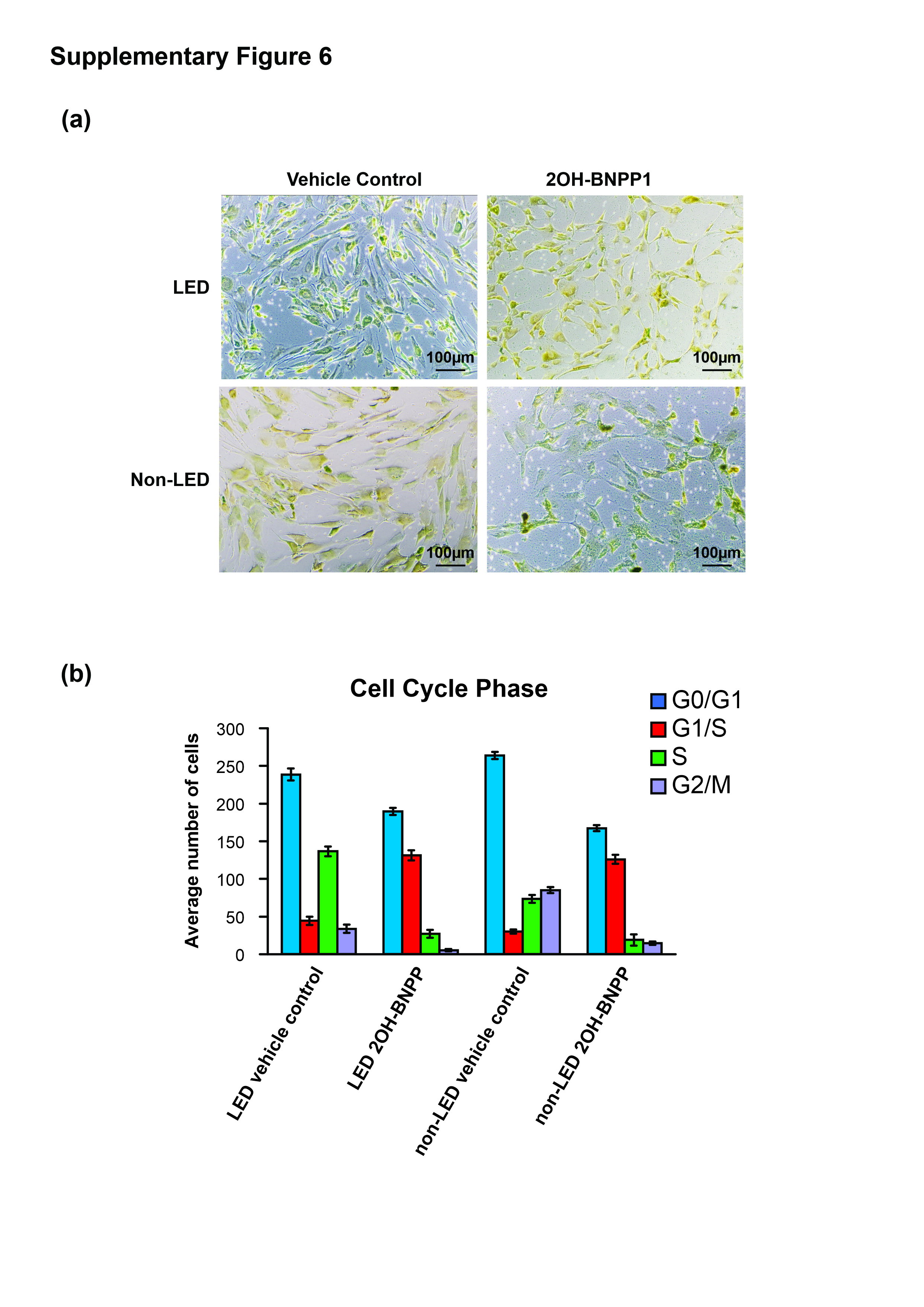

Supplement: Supplementary file 14 — Supplementary Figure 6 [file 41366_2021_1002_MOESM14_ESM.tif]
